# EGF-induced nuclear translocation of SHCBP1 promotes bladder cancer progression through inhibiting RACGAP1-mediated RAC1 inactivation

**DOI:** 10.1038/s41419-021-04479-w

**Published:** 2022-01-10

**Authors:** Hubin Yin, Chen Zhang, Zongjie Wei, Weiyang He, Ning Xu, Yingjie Xu, Tinghao Li, Ke Ren, Youlin Kuang, Xin Zhu, Fangchao Yuan, Haitao Yu, Xin Gou

**Affiliations:** 1 Department of Urology, The First Affiliated Hospital of Chongqing Medical University, Chongqing, 400016 China; 2 Chongqing Key Laboratory of Molecular Oncology and Epigenetics, The First Affiliated Hospital of Chongqing Medical University, Chongqing, 400016 China; 3 Department of Obstetrics and Gynecology, The First Affiliated Hospital of Chongqing Medical University, Chongqing, 400016 China; 4 Departments of Urology, The First Affiliated Hospital of Fujian Medical University, Fuzhou, PR China; 5 Central Laboratory, The First Affiliated Hospital of Chongqing Medical University, Chongqing, 400016 China

**Keywords:** Bladder cancer, RHO signalling

## Abstract

Bladder cancer is a highly heterogeneous and aggressive malignancy with a poor prognosis. EGF/EGFR activation causes the detachment of SHC-binding protein 1 (SHCBP1) from SHC adapter protein 1 (SHC1), which subsequently translocates into the nucleus and promotes cancer development via multiple signaling pathways. However, the role of the EGF-SHCBP1 axis in bladder cancer progression remains unexplored. Herein, we report that SHCBP1 is upregulated in bladder cancer tissues and cells, with cytoplasmic or nuclear localization. Released SHCBP1 responds to EGF stimulation by translocating into the nucleus following Ser273 phosphorylation. Depletion of SHCBP1 reduces EGF-induced cell migration and invasiveness of bladder cancer cells. Mechanistically, SHCBP1 binds to RACGAP1 via its N-terminal domain of amino acids 1 ~ 428, and this interaction is enhanced following EGF treatment. Furthermore, SHCBP1 facilitates cell migration by inhibiting RACGAP-mediated GTP-RAC1 inactivation, whose activity is indispensable for cell movement. Collectively, we demonstrate that the EGF-SHCBP1-RACGAP1-RAC1 axis acts as a novel regulatory mechanism of bladder cancer progression, which offers a new clinical therapeutic strategy to combat bladder cancer.

## Introduction

Bladder cancer is the 10th most commonly diagnosed cancer in the world, with an estimated 573,278 new cases and 212,536 deaths [1]. Approximately 25% of bladder cancer patients are diagnosed with muscle‐invasive bladder cancer (MIBC), which has a high risk of distant metastasis and mortality [2]. Uncovering the molecular mechanisms underlying bladder cancer progression and identifying novel prognostic factors that can better identify suitable clinical regimens for patients are urgent issues.

SHC SH2 domain-binding protein 1 (SHCBP1), a member of the Src homolog and collagen homolog (SHC) family, plays an essential role in the regulation of multiple signal transduction pathways, such as EGF/EGFR, FGF, NF-κB, MAPK/ERK, PI3K/Akt, TGF-β1/Smad and Wnt/β-catenin [3]. Increasing evidence has shown that SHCBP1 is involved in the occurrence and development of various types of tumors, accelerating the progression of diseases and facilitating the growth and maintenance of a tumorigenic state. Our previous study revealed that SHCBP1 has a tumor-promoting function in prostate cancer cells, probably through suppression of LATS1 and TP53. Furthermore, SHCBP1 was significantly upregulated in prostate cancer tissues compared with BPH tissues [4]. Interestingly, SHCBP1 also mediates EGF-induced activation of β-catenin signaling in non-small cell lung carcinoma (NSCLC) cells. SHCBP1 can translocate to the nucleus following EGF stimulation, induce the interaction between β-catenin and CBP, directly increase the transactivating activity of β-catenin, and enhance the development of stemness properties of NSCLC cells [5]. However, the role of SHCBP1 in the progression of bladder cancer has not been reported.

In the present study, we explored the clinical significance and regulatory mechanisms of SHCBP1 in bladder cancer. We found that SHCBP1 was upregulated in human bladder cancer tissues compared with adjacent urothelial mucosa tissues. Importantly, translocation of SHCBP1 to the nucleus induced by EGF treatment promoted the cell migration and invasiveness of bladder cancer cells in vitro and in vivo. Moreover, EGF stimulation increased the interaction between SHCBP1 and RACGAP1 in the nucleus, which attenuated the catalytic activity of RACGAP1 toward GTP-RAC1, an active protein that sustains epithelial cell polarization, cell movement, and cytoskeletal reorganization.

## Results

### SHCBP1, an SHC1-binding protein, is upregulated in bladder cancer tissues and cells

**Fig. 1:**
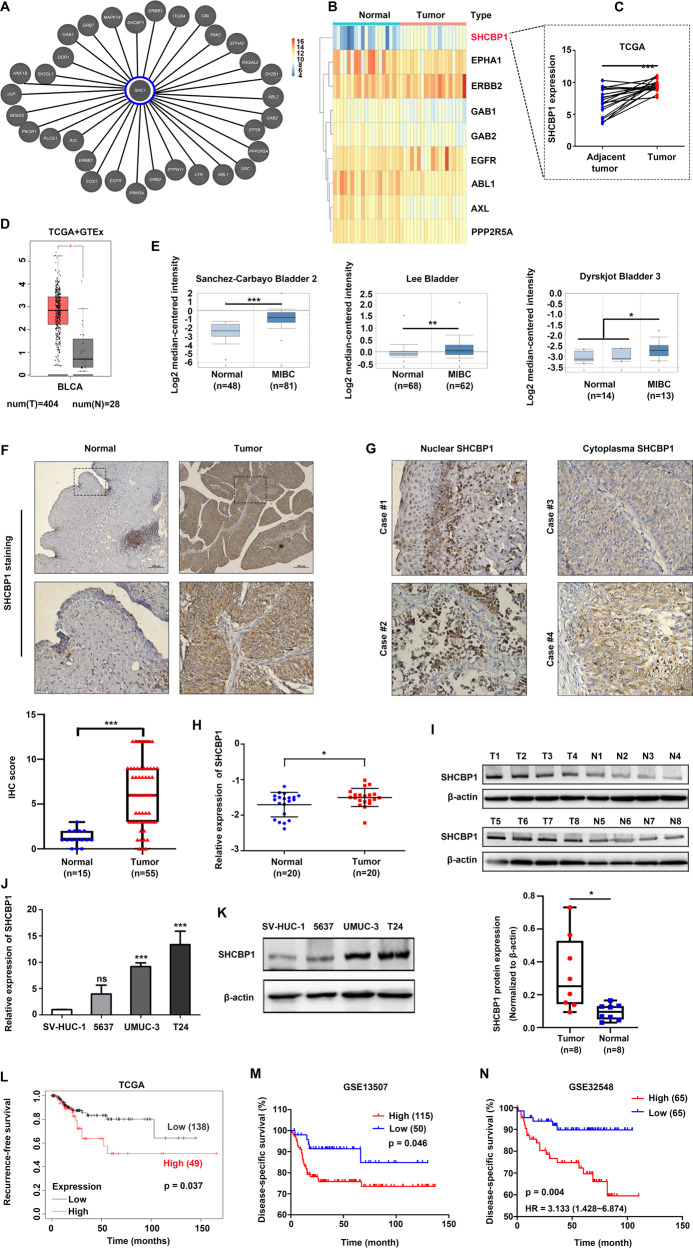
SHCBP1 is overexpressed in bladder cancer. **A** Thirty-two SHC1-binding proteins are shown. **B** Heat map representation showing the members among 32 genes differentially expressed between 19 cases of bladder cancer and paired adjacent nontumor tissues from the TCGA dataset, fold change = 2, *p* < 0.05. **C** SHCBP1 expression in 19 cases of bladder cancer and paired adjacent tissues from the TCGA dataset. **D** SHCBP1 expression in the TCGA and GTEx databases. **E** SHCBP1 expression in three cohorts (Sanchez-Carbayo Bladder 2, Lee Bladder, and Dyrskjot Bladder 3) from the Oncomine database. **F** The expression of SHCBP1 in bladder cancer and normal tissues was detected by IHC. Representative images and IHC scores are shown, ***p < 0.001. **G** Representative IHC images of the subcellular location of the SHCBP1 protein. **H** RT-qPCR analysis of SHCBP1 expression in twenty cases of bladder cancer and normal tissues; *p < 0.05. **I** Immunoblotting analysis of SHCBP1 expression in eight cases of bladder cancer and normal tissues. N nonneoplastic bladder tissues, T tumor tissue, *p < 0.05. **J**, **K** RT-qPCR and immunoblotting analysis of SHCBP1 expression in three bladder cancer cell lines (5637, T24, and UMUC-3) and ureteral epithelial cells (SV-HUC-1). **L** Kaplan–Meier curves depicting RFS in high‑ and low‑SHCBP1 patients from TCGA dataset. **M**, **N** Kaplan–Meier analysis of the relationship between SHCBP1 expression and DSS in the GSE13507 and GSE32548 cohorts.

A previous study showed that SHC1 facilitated the development of bladder cancer [6]. To gain insight into the subset of SHC1-binding proteins and identify the key factor for accelerating tumor progression, we mapped 32 binding proteins associated with SHC1 according to the findings of Wengui Shi et al. [7] and screened for candidates among 32 genes that were differentially expressed between 19 paired bladder cancer and adjacent nontumor tissues (Fig. 1A, B). By analyzing The Cancer Genome Atlas (TCGA) dataset, we found that SHCBP1 expression was predominantly upregulated in bladder cancer samples as compared with corresponding nontumor tissues (Fig. 1C). Data from GEPIA also showed that the expression of SHCBP1 in tumor tissues was higher than that in normal tissues (Fig. 1D). Additionally, three cohorts (Sanchez-Carbayo Bladder 2, Lee Bladder, and Dyrskjot Bladder 3) from the Oncomine database supported this finding: compared to normal samples, SHCBP1 mRNA was significantly increased in MIBC samples (Fig. 1E). The IHC results showed that SHCBP1 staining was stronger in bladder cancer tissues than in adjacent normal tissues (Fig. 1F), and SHCBP1 was observed in the cytoplasm and nucleus (Fig. 1G). RT-qPCR revealed that SHCBP1 expression was significantly elevated in 20 bladder cancer tissues, compared with 20 adjacent normal tissues (Fig. 1H). Immunoblotting was used to determine the protein expression of SHCBP1 in tissue samples, and the results showed that SHCBP1 protein expression was also higher in tumors than in normal tissues (Fig. 1I). Consistent with this finding, SHCBP1 expression was increased in T24 and UMUC-3 cells at both the protein and mRNA levels compared to ureteral epithelial SV-HUC-1 cells (Fig. 1J, K).

The TCGA cohort and two GEO cohorts (GSE13507 and GSE32548) were used to analyze the relationship between SHCBP1 expression and prognosis. Kaplan–Meier curves showed that patients with high SHCBP1 expression had a significantly shorter recurrence-free survival (RFS) or disease-specific survival (DSS) than those with low SHCBP1 expression (Fig. 1L–N).

### SHCBP1 translocates into the nucleus following EGF stimulation

**Fig. 2:**
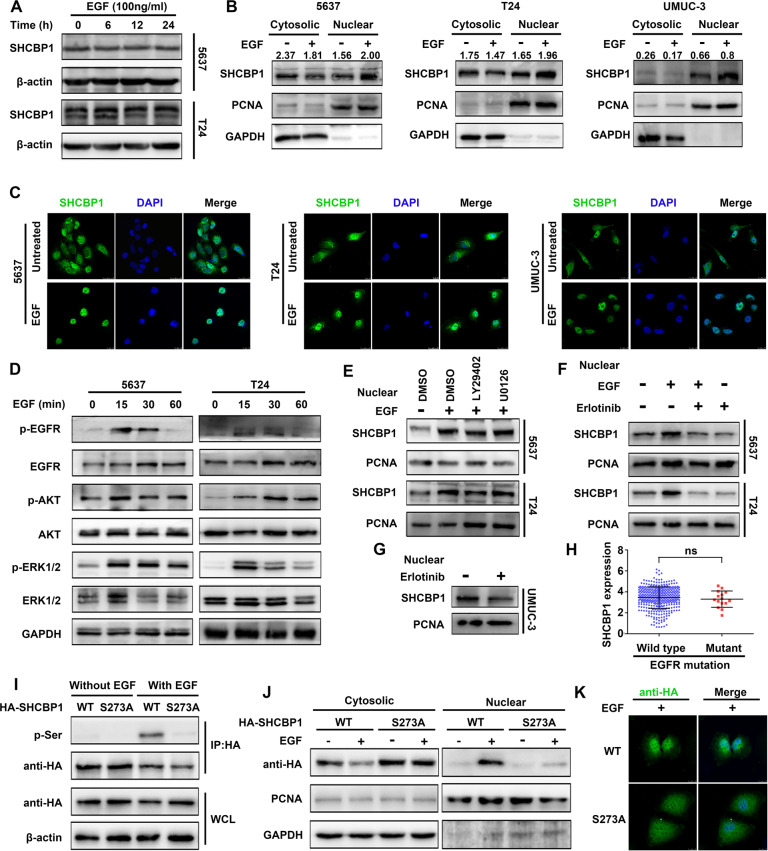
EGF promotes SHCBP1 nuclear translocation. **A** Immunoblotting analysis showing total expression of SHCBP1 protein after EGF treatment (100 ng/ml) at the indicated time points in 5637 and T24 cells. **B** Immunoblotting analysis showing SHCBP1 redistribution in the cytoplasm and nucleus following EGF stimulation for 30 min in 5637, T24, and UMUC-3 cells. Quantified value of SHCBP1 compared to internal control was shown. **C** IF images indicating SHCBP1 localization from the cytoplasm to the nucleus after EGF treatment in 5637, T24, and UMUC-3 cells. **D** Immunoblotting analysis showing the expression of p-EGFR, p-AKT, and p-ERK1/2 in response to EGF at the indicated time points in 5637 and T24 cells. **E** Immunoblotting analysis determining the impact of a PI3K/Akt inhibitor (LY294002, 20 µM) or an ERK1/2 inhibitor (U0126, 10 µM) on the nuclear translocation of SHCBP1 in 5637 and T24 cells. **F** EGF-induced SHCBP1 nuclear translocation was examined after erlotinib (EGFR tyrosine kinase inhibitor) treatment in 5637 and T24 cells. **G** SHCBP1 nuclear translocation was examined by immunoblotting in the absence or presence of erlotinib in UMUC-3 cells. **H** SHCBP1 expression in bladder cancer patients with or without EGFR mutation was compared (data from TCGA). **I** Following immunoprecipitation against HA, SHCBP1 phosphorylation was detected using immunoblotting with an anti-phosphoserine (pSer) antibody in lysates from T24 cells transfected with HA-SHCBP1 (WT) or mutant HA-SHCBP1 (S273A) and treated with EGF. **J**, **K** Immunoblotting and IF showing the subcellular localization of HA-SHCBP1 or mutant HA-SHCBP1 (S273A) in T24 cells in response to EGF.

EGF acts through EGFR to facilitate the development of various types of cancers, including urothelial carcinoma [8]. A previous study demonstrated EGF-induced SHCBP1 dissociation from SHC1 and nuclear translocation. SHCBP1 protein in the nucleus directly promotes the transactivating activity of β-catenin, consequently leading to the development of NSCLC cell stemness and malignant evolution [5]. Herein, we wondered whether SHCBP1 responds to EGF/EGFR activation through translocation to the nucleus in bladder cancer. First, we found that EGF did not increase the total expression of SHCBP1 protein (Fig. 2A) but increased SHCBP1 expression in the nuclei of three bladder cancer cell lines, as revealed by immunoblotting and IF (Fig. 2B, C). EGF activated the EGFR, AKT, and ERK1/2 pathways in a time-dependent manner (Fig. 2D). Blockade of p-AKT and p-ERK1/2 using specific inhibitors had no effect on SHCBP1 nuclear localization induced by EGF in 5637 and T24 cells (Fig. 2E). However, this event could be abrogated by preincubation with erlotinib, an EGFR tyrosine kinase inhibitor (Fig. 2F). Erlotinib treatment also decreased SHCBP1 distribution in the nucleus in UMUC-3 cells (Fig. 2G), in addition, there is no difference in SHCBP1 expression between the EGFR wild-type group and the EGFR mutant group in the TCGA dataset, suggesting that no correlation existed between SHCBP1 expression and EGFR mutation (Fig. 2H). Wengui Shi [9] found that phosphorylation at the S273 site was indispensable for SHCBP1 nuclear localization. Hence, we constructed a mutant SHCBP1 (SHCBP1^S273A^) and detected SHCBP1 phosphorylation using an antibody against phosphoserine. Mutagenesis analysis showed that EGF stimulation induced serine phosphorylation in the wildtype but to a lesser extent in the mutant (Fig. 2I). Immunoblotting and IF results indicated that the S273A mutant significantly impeded EGF-induced SHCBP1 nuclear localization in T24 cells (Fig. 2J). These findings suggest that enhanced nuclear translocation of SHCBP1 in response to EGF was dependent on EGFR tyrosine kinase signaling and phosphorylation at the S273 site but not on the ERK1/2 or PI3K/AKT pathway.

### SHCBP1 mediates EGF-induced migration and invasion of bladder cancer cells

**Fig. 3:**
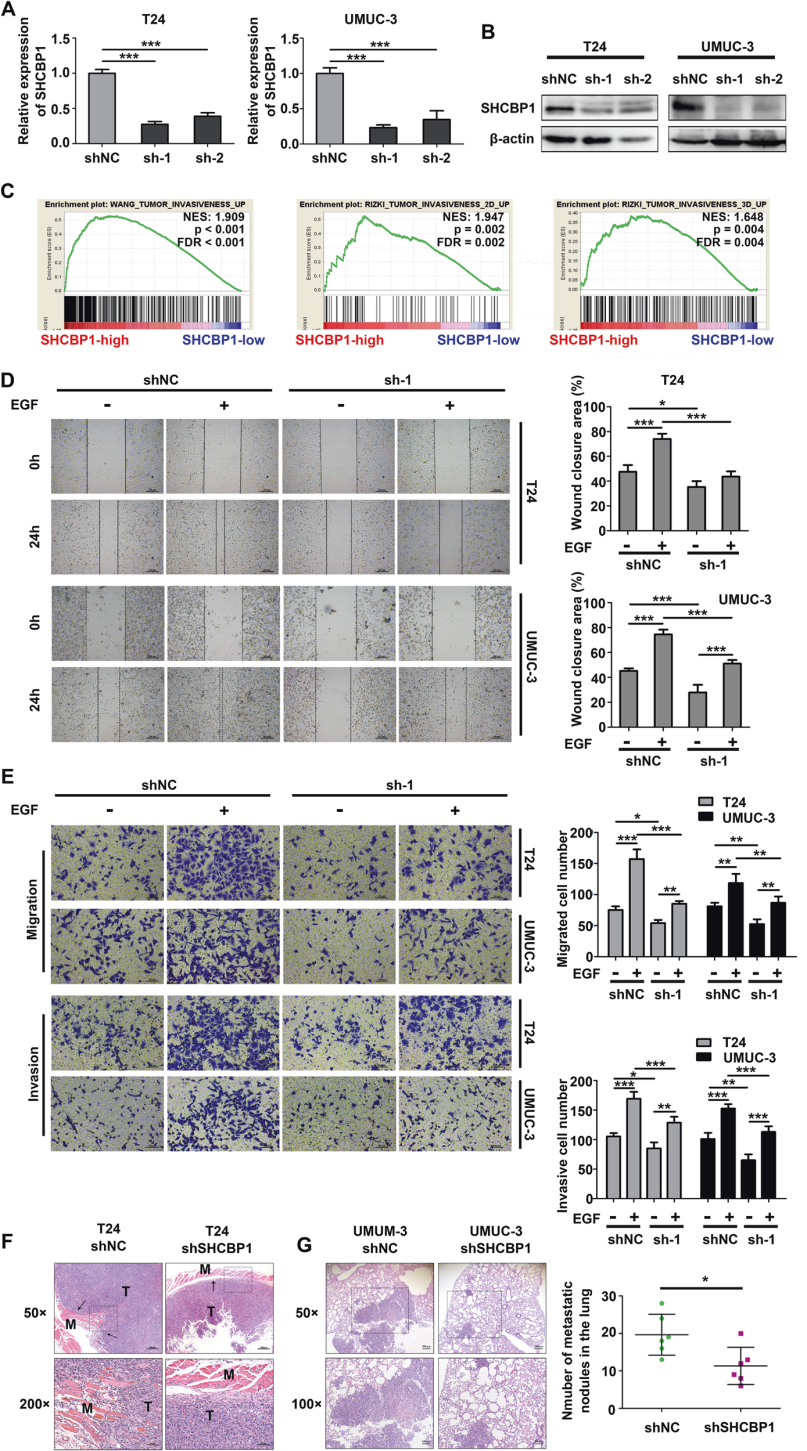
SHCBP1 is involved in EGF-induced cell migration and invasiveness in bladder cancer. **A**, **B** RT-qPCR and immunoblotting analysis showing SHCBP1 expression in the indicated cells transfected with shNC or shSHCBP1. **p < 0.01 and ***p < 0.001. **C** GSEA indicating that SHCBP1 expression was positively associated with tumor invasiveness (data from TCGA). **D** Scratch assays indicating the effect of SHCBP1 depletion on EGF-induced motility of T24 and UMUC-3 cells. *p < 0.05 and ***p < 0.001. **E** Transwell assays showing the effect of SHCBP1 depletion on EGF-induced migration and invasion of T24 and UMUC-3 cells. *p < 0.05, **p < 0.01 and ***p < 0.001. **F** H&E staining of both the tumor tissue and muscle tissue in the subcutaneous tumors xenografted with the indicated cells. Black arrows point to the direction of tumor invasiveness. **G** H&E staining of metastatic lesions in the lungs of mice injected with the indicated cells for 6 weeks. The number of metastatic nodules was calculated for each group (*n* = 6) in the panel. *p < 0.05.

The motility and invasiveness of bladder cancer cells have been shown to be promoted by EGF stimulation [10, 11]. Together with the aforementioned effect of EGF on SHCBP1 redistribution, as shown above, we then asked whether SHCBP1 is involved in mediating the EGF-induced malignant behaviors of bladder cancer cells. We stably knocked down SHCBP1 in UMUC-3 and T24 cells using two human shRNA sequences cloned into the lentiviral vector, and SHCBP1 expression was significantly reduced at both the mRNA and protein levels in T24 and UMUC-3 cells (Fig. 3A, B), indicating the great interfering effect of shRNA on the target.

Gene set enrichment analysis (GSEA) based on the TCGA-BLCA cohort was performed to explore the relative biological processes. The results showed that TUMOR INVASIVENESS was mainly enriched in the SHCBP1-high group (Fig. 3C). Inhibiting endogenous SHCBP1 expression significantly repressed the EGF-triggered motility of T24 and UMUC-3 cells in wound healing and Transwell (without Matrigel) assays (Fig. 3D, E and Supplementary Fig. 1). Matrigel-coated Transwell chambers were used to detect the effect of SHCBP1 depletion on tumor cell invasion. Consistent with the cell migration assays, SHCBP1 loss attenuated EGF-enhanced cell invasion in T24 and UMUC-3 cells (Fig. 3E and Supplementary Fig. 1). In an in vivo study, tumor cells in each xenograft inoculated with shNC T24 cells produced invasion fronts and irregularly invaded deep muscle tissues, while most xenografts infected with shSHCBP1 T24 cells presented a clear tissue boundary between the tumor and muscle (Fig. 3F). Moreover, Balb/c nude mice were injected via the tail vein with UMUC-3 cells stably transfected with shSHCBP1 or shNC. Images of H&E staining of lung tissues were observed under a microscope, and the results showed that the mice in the control group had more metastatic nodules in the lungs than those in the shSHCBP1 group (Fig. 3G). These findings suggested that SHCBP1 plays an essential role in regulating the EGF-induced migration and invasiveness of bladder cancer cells.

### SHCBP1 mediates the cell proliferation of bladder cancer cells

The role of SHCBP1 in proliferation of bladder cancer cells remains unclear. We herein performed experiments to study whether SHCBP1 affects proliferation phenotype of bladder cancer cells. CCK8 and EdU assays showed that depletion of SHCBP1 significantly suppressed cell growth activity DNA synthesis (Supplementary Fig. 2A, B). IHC results showed that the staining intensity of Ki67 was weaker in shSHCBP1 group that in control group (Supplementary Fig. 2C), suggesting an impaired growth activity. We next investigate whether RACGAP1-RAC1 involves in SHCBP1-mediated cell proliferation. As shown in Supplementary Fig. 2D, ectopic expression of RACGAP1 can rescue the loss of SHCBP1 caused decreased cell proliferation rate, however, blockage of GTP-RAC1 using EHop-016 did not alter the positive effect of RACGAP1 on cell growth, suggesting SHCBP1-RACGAP1 regulates cell proliferation in a RAC1-independent manner.

### RACGAP1 is an interactive factor of SHCBP1

**Fig. 4:**
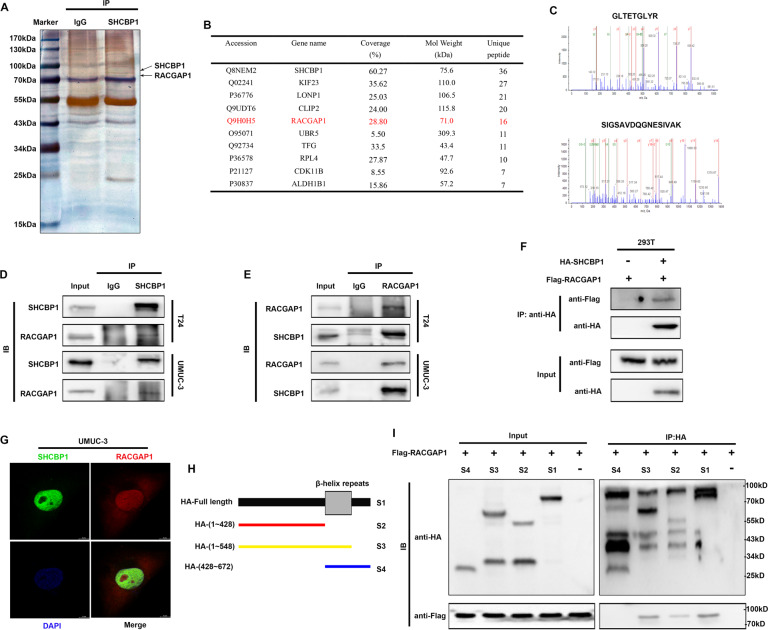
RACGAP1 is an interactive protein of SHCBP1. **A** Endogenous SHCBP1 was immunoprecipitated with an anti-SHCBP1 antibody, and silver staining was performed to visualize the proteins interacting with SHCBP1. **B** Distinct protein bands in the GEL were subjected to mass spectrometry, and the top ten interacting partners are shown. **C** The peptide map of the protein band marked by the arrow is identified as RACGAP1. **D**, **E** The endogenous interaction between SHCBP1 and RACGAP1 was detected by coimmunoprecipitation and immunoblotting in T24 and UMUC-3 cells. **F** The exogenous interaction between HA-SHCBP1 and Flag-RACGAP1 was assessed by coimmunoprecipitation and immunoblotting in 293 T cells. **G** Confocal fluorescence microscopy observing the colocalization of SHCBP1 and RACGAP1 in UMUC-3 cells. **H**, **I** Immunoprecipitation and immunoblotting analysis indicated that the N-terminal amino acid sequence (1 ~ 428) of SHCBP1 specifically interacted with RACGAP1.

To identify unknown binding partners of SHCBP1, whole-cell protein lysates from T24 cells were extracted and immunoprecipitated with SHCBP1 or IgG antibodies. Silver staining was then performed to visualize differential protein bands in the precipitated product, as shown in Fig. 4A. Two groups of whole-cell lysates were further analyzed by liquid chromatography-mass spectrometry, and Fig. 4B shows the top ten differentially expressed proteins. According to the results, RACGAP1 was found to be the major partner of the SHCBP1 complex (Fig. 4A, B), and its peptides spectrogram is shown in Fig. 4C.

Given the significant role of RACGAP1 in oncogenic development and tumor recurrence [9, 12], the regulatory mechanism related to RACGAP1 is well worthy of attention. Therefore, we selected RACGAP1 for the subsequent study. Endogenous binding between SHCBP1 and RACGAP1 was confirmed by coimmunoprecipitation and immunoblotting assays in T24 and UMUC-3 cells (Fig. 4D, E). HA-SHCBP1 and Flag-RACGAP1 vectors were transfected into HEK-293T cells to detect the exogenous interaction between both proteins, and the results indicated that HA-SHCBP1 interacted with Flag-RACGAP1 in HEK-293T cells (Fig. 4F). The colocalization of SHCBP1 and RACGAP1 was observed in the nucleus of UMUC-3 cells using an immunofluorescent staining assay (Fig. 4G). Moreover, to identify the SHCBP1 domain responsible for its binding with RACGAP1, HA-tagged serially truncated SHCBP1 constructs [full-length (1 ~ 672), amino acid sequences from 1 to 428 (1 ~ 428), 1 ~ 548 and 428 ~ 672, shown in Fig. 4H] and Flag-RACGAP1 were cotransfected into HEK-293T cells. An N-terminal domain of 1 ~ 428 in SHCBP1 was shown to be pivotal for its binding with RACGAP1 (Fig. 4I).

### EGF stimulation enhanced the interaction between SHCBP1 and RACGAP1

**Fig. 5:**
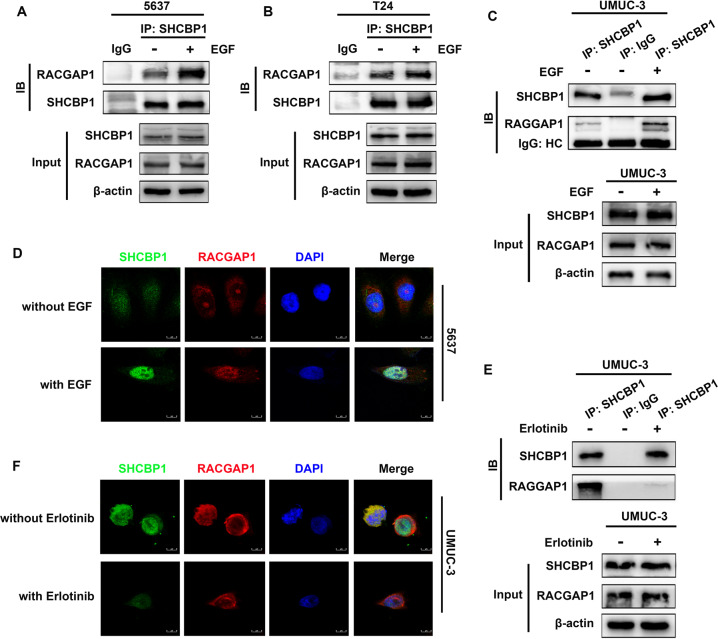
EGF enhances the interaction between SHCBP1 and RACGAP1. **A, B, C** Immunoprecipitation and immunoblotting assays were conducted to evaluate the effect of EGF on the binding of SHCBP1 and RACGAP1 in 5637, T24, and UMUC-3 cells. HC: Heavy chain. **D** An IF assay was used to observe the subcellular location of SHCBP1 and RACGAP1 in 5637 cells in response to EGF. **E** The effect of erlotinib on the binding of SHCBP1 and RACGAP1 in UMUC-3 cells was examined using immunoprecipitation and immunoblotting assays. **F** IF assay was used to observe the subcellular location of SHCBP1 and RACGAP1 following erlotinib treatment.

RACGAP1 is a component of the central spindlin complex in the nucleus, and EGF can facilitate the accumulation of nuclear SHCBP1 protein. Accordingly, we further asked whether EGF is involved in the regulation of the binding between SHCBP1 and RACGAP1. As shown in Fig. 5A, B, C, the binding between SHCBP1 and RACGAP1 was enhanced in response to EGF in T24, 5637, and UMUC-3 cells, without alteration of the total protein expression of SHCBP1 and RACGAP1, and EGF stimulation induced SHCBP1 and RACGAP1 to colocalize in the nucleus in 5637 cells (Fig. 5D). The suppressive activity of EGFR using erlotinib impeded the interaction and colocalization between SHCBP1 and RACGAP1 in UMUC-3 cells (Fig. 5E, F), indicating that EGF-EGFR signaling plays an essential role in maintaining their binding.

### SHCBP1 inhibits RACGAP-mediated hydrolysis of GTP-RAC1

**Fig. 6:**
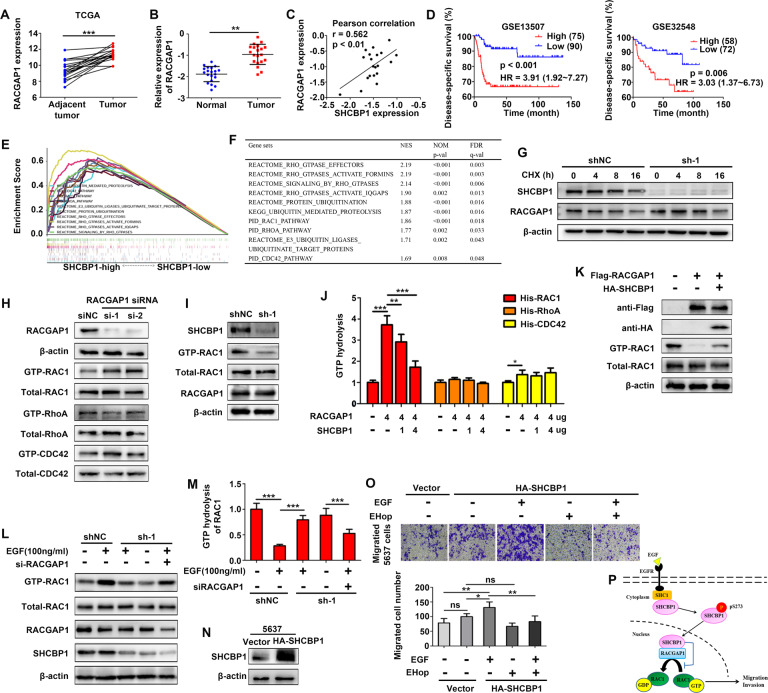
SHCBP1 suppresses the GAP activity of RACGAP to GTP-RAC1. **A** RACGAP1 expression in 19 cases of bladder cancer and paired adjacent tissues (data from TCGA). ***p < 0.001. **B** RT-qPCR analysis of RACGAP1 expression in twenty cases of bladder cancer and normal tissues; **p < 0.01. **C** Correlation between SHCBP1 and RACGAP1 mRNA expression in bladder cancer tissues. **D** Kaplan–Meier analysis of the relationship between RACGAP1 expression and DSS in the GSE13507 and GSE32548 cohorts. **E**, **F** GSEA based on the TCGA dataset showed that the indicated gene sets, including Rho-GTPase signaling and ubiquitination processes, were enriched in the SHCBP1-high group. NES: normalized enrichment score; FDR: false discovery rate. **G** Immunoblotting assay to assess the impact of SHCBP1 inhibition on RACGAP1 protein expression in T24 cells treated with cycloheximide (40 nM) at the indicated time points. **H** Pull-down and immunoblotting assays of the GTPase activity of RAC1 and RhoA after knockdown of RACGAP1 in T24 cells. **I** Pull-down and immunoblotting assays of the GTPase activity of RAC1 after SHCBP1 depletion in T24 cells. **J** The RACGAP1-mediated hydrolysis activity toward small GTPases was tested in T24 cells transfected with certain concentrations of HA-SHCBP1 and Flag-RACGAP1 in an in vitro GAP assay. *p < 0.05, **p < 0.01 and ***p < 0.001. **K** Immunoblotting analysis indicating the alteration of active RAC1 following Flag-RACGAP1 and HA-SHCBP1 ectopic expression in T24 cells. **L** Pull-down assays for RAC1 activity were prepared in shNC-, shSHCBP1- or RACGAP1 siRNA-treated T24 cells in the presence or absence of EGF, and the expression of the indicated proteins was analyzed by immunoblotting. **M** The GTP hydrolysis activity toward RAC1 was measured in shNC-, shSHCBP1- or RACGAP1 siRNA-treated T24 cells in the presence or absence of EGF in an in vitro GAP assay. ***p < 0.001. **N** Immunoblotting analysis showing SHCBP1 expression in 5637 cells transfected with HA-SHCBP1. **O** Transwell assays showing the effect of HA-SHCBP1 ectopic expression and EHop-016 (10 μM) on EGF-induced migration of 5637 cells. *p < 0.05, **p < 0.01, ns: not significant. **P** A schematic diagram of SHCBP1 promoting EGF-stimulated cell migration and invasiveness by reducing the hydrolysis activity of RACGAP toward GTP-RAC1 is shown.

RACGAP1 functions as an oncogenic protein in bladder cancer [13]. Compared with adjacent nontumor tissues, RACGAP1 expression was obviously upregulated in 19 paired bladder cancer tissues in the TCGA cohort (Fig. 6A). We then examined its expression in 20 pairs of fresh bladder cancer and normal bladder tissues. RT-qPCR confirmed that RACGAP1 expression was elevated in bladder cancer tissues (Fig. 6B), and there was a significant positive correlation between SHCBP1 and RACGAP1 mRNA expression (Fig. 6C). Kaplan–Meier curves for disease-specific survival in patients with bladder cancer were plotted according to the relative expression levels of RACGAP1 of two GEO cohorts (GSE13507 and GSE32548), and the results indicated that patients with RACGAP1-high had poorer disease-specific survival than those with RACGAP1-low (Fig. 6D).

To explore the mechanism underlying SHCBP1-involved malignant behaviors in bladder cancer, GSEA was performed using TCGA-BLCA RNA-sequencing data. Upregulation of SHCBP1 correlated significantly with Rho-GTPase signaling and ubiquitination processes (Fig. 6E, F).

Since RACGAP1 is a GTPase-activating protein (GAP) that can return Rho GTPases to an inactive and GDP-bound state and SHCBP1 alternation did not influence the stability of RACGAP1 protein following cycloheximide (CHX) treatment (Fig. 6G), we were prompted to determine whether SHCBP1 can be involved in regulating the RACGAP-mediated activity of Rho GTPases. As expected, we found that knockdown of RACGAP1 expression can activate RAC1 protein by increasing the level of GTP-RAC1. GTP-RhoA and GTP-CDC42, the active forms of RhoA and CDC42, respectively, were unchanged in response to RACGAP1 loss (Fig. 6H). Moreover, suppression of SHCBP1 reduced the expression of GTP-RAC1 but not RACGAP1 in UMUC-3 cells (Fig. 6I), suggesting a potential regulatory effect of SHCBP1 on GAP activity.

We next performed an in vitro GAP assay, and the results showed that RACGAP1 exhibited marked GAP activity toward RAC1 and slight activity toward CDC42 (Fig. 6J). Interestingly, SHCBP1 impaired the RACGAP-stimulated GTP hydrolysis activity of RAC1 in a concentration-dependent manner (Fig. 6J), and HA-SHCBP1 rescued the Flag-RACGAP-mediated inactivation of RAC1 in T24 cells (Fig. 6K). In addition, EGF treatment increased GTP-RAC1 expression and attenuated the RACGAP-mediated GTP hydrolysis activity of RAC1, but this effect was reversed after SHCBP1 loss and was reproduced after RACGAP1 knockdown in T24 cells (Fig. 6L, M), suggesting an indirect regulatory effect of the EGF/SHCBP1 axis on RAC1 activity. Subsequently, 5637 cells were transfected with HA-SHCBP1, and protein expression was detected using anti-SHCBP1 (Fig. 6N). EGF facilitated the migration of 5637 cells expressing SHCBP1, but elimination of RAC1 activity using EHop-016, a specific RAC1 inhibitor, decreased EGF-induced migration in 5637-SHCBP1 cells (Fig. 6O). These findings indicated that the EGF-SHCBP1 axis rescued the RACGAP1-mediated inactivation of RAC1, thus promoting cell motility. A diagrammatic sketch of the mechanism was drawn and is shown in Fig. 6P.

## Discussion

The SHC adapter protein has been considered an SH2-containing proto-oncogene involved in mediating diverse signaling pathways. The SHC1 scaffold drives the temporal flow of signaling information in response to EGF through multiple waves of Ser/Thr phosphorylation events and protein interactions [14]. Here, we identified an SHC1-binding protein, SHCBP1, which was overexpressed and related to poor DSS in patients with bladder cancer. SHCBP1 binds to Shc1 prior to EGF stimulation but is released and translocates into the nucleus after EGF treatment, while other SHC1-binding partners show increased affinity with SHC1 in response to EGFR activation, suggesting that nuclear translocation of SHCBP1 may play a role distinct from other SHC1-binding proteins in EGF/EGFR-mediated signal transduction [5]. In addition, nuclear translocation of SHCBP1 is a downstream consequence of HER2 activation, which is dependent on phosphorylation of SHCBP1 at the Ser273 site. Blocking HER2 activation using trastuzumab effectively abolished EGF-induced nuclear localization of SHCBP1 in gastric cancer cells [7]. In our study, we confirmed that upon EGF induction, SHCBP1 translocates to the nucleus, where it binds to RACGAP1 through its N-terminal domain of amino acids 1 ~ 428, and inhibits the GAP activity of RACGAP1 toward RAC1, causing accelerated EGF-induced cell spreading and increased invasiveness of bladder cancer cells. Our findings facilitate a deeper understanding of EGF-evoked intracellular signaling networks and the regulatory mechanism of active RAC1 involved in SHCBP1 and RACGAP1.

SHCBP1 is closely associated with multiple biological processes and plays an essential role in signaling pathways. Wengui Shi et al [7] reported that SHCBP1 translocated into the nucleus and interacted with PLK1 for cell mitosis, and they also proved that SHCBP1 can locate at the midbody. Asano et al. [15] reported that SHCBP1 interacted with RACGAP1 at midbody for cytokinesis. That means SHCBP1 is an essential regulator of mitosis following detached from Shc and translocated into the nucleus. During cytokinesis, SHCBP1 binding to central spindlin facilitates midbody organization and the completion of abscission [16]. RACGAP1 impedes active RAC1 via its GAP domain and induces furrow ingression in mammalian cells, while SHCBP1 inhibits RACGAP1-mediated inactivation of RAC1. The Aurora-B-mediated phosphorylation of SHCBP1 at the S634 site attenuates the interaction of SHCBP1 with RACGAP1 and prevents the premature localization of SHCBP1 to the central spindle, ensuring that RAC1 inactivation caused by RACGAP promotes the ingression of the cytokinetic furrow during early anaphase [15], which is consistent with our finding that SHCBP1 suppresses RACGAP-mediated hydrolysis of GTP-RAC1 but not RhoA and CDC42 in bladder cancer cells, leading to the accumulation of active RAC1.

Biological processes coordinated by RAC1 in tumor cells are achieved through the regulation of RAC1 activity and the spatiotemporal activation of RAC1, switching between active and inactive states at different subcellular locations, including the nucleus, plasma membrane, and mitochondria [17]. The nuclear localization of RAC1 depends on the cell cycle. Accumulated RAC1 is observed in the nucleus in the late G2 phase, and a decrease in RAC1 in the nucleus occurs during the early G1 phase [18]. In addition to the regulation of cytokinesis, RAC1 is involved in cytoskeletal rearrangement and cell motility. Nuclear accumulation of RAC1 promotes nuclear plasticity, causing a loss of cytoplasmic active RAC1 with a concomitant increase in RhoA that drives actomyosin-mediated changes in cell morphology, the two attributes contribute to intensifying the aggressiveness of tumor cells [19]. Nuclear RAC1 in complex with β-catenin facilitates RAC1-dependent phosphorylation of β-catenin on serines 161 and 605, resulting in the formation of nuclear β-catenin-LEF-1, which is necessary for the transactivation of Wnt-dependent genes [20]. Our results showed that blockade of GTP-RAC1 using Ehop-016 inverses EGF-induced cell migration of 5637 cells expressing SHCBP1, indicating its positive effect on cell movement in bladder cancer, which is supported by other studies. The RAC1 short-3' UTR isoform promotes the oncogenic and metastatic capacities in urothelial bladder carcinoma cells both in vitro and in vivo [21]. Treatment with 200 nM MBQ-167, a RAC1 inhibitor, limited the enhanced migration, invasion, and filopodia formation resulting from KDM6A depletion in T24 cells [22]. The findings above suggest that the movement of bladder cancer cells is at least partially dependent on the activity of RAC1.

Elevated RACGAP1 expression has been recorded in various cancers, including hepatocellular carcinoma [23], basal-like breast cancer [24], and gallbladder cancer [25]. RACGAP1 stimulates STAT3 phosphorylation and promotes its nuclear translocation, thus facilitating cell proliferation, migration, and decreased chemosensitivity to doxorubicin in bladder cancer [13]. RACGAP1 has previously been shown to exert GAP activity toward RAC1 and CDC42 but not RhoA [26]. The FLNa–IQGAP1 complex assembling at β1-integrin activation sites recruits RACGAP1 to the cell periphery to suppress RAC1 activity during cell spreading on fibronectin [27]. However, depletion of RACGAP1 enhanced the random migration velocity of SUM149 cells, with an increase in GTP-bound RhoA but not RAC1 or CDC42 [24]. In this study, we found that cell movement is RAC1-dependent in bladder cancer, and RAC1 activity, but not RhoA and CDC42, is inhibited by RACGAP1, which is partially reversed following SHCBP1 ectopic expression. Considering the oncogenic role of RACGAP1 in the malignant behaviors of bladder cancer cells, we inferred that RACGAP1 exerts cancer-promoting functions via the upregulation of p-STAT3 [28], active RhoA, and p-ERK1/2 [29]. In addition, RACGAP1 can also inactivate RAC1 by its GAP activity to play a tumor suppressor role, but the comprehensive effect of the two forces is to drive cancer development, indicating that upon EGF induction, SHCBP1 promotes cell motility through inhibition of the tumor suppressor effect of RACGAP1 in bladder cancer.

In summary, our research has identified SHCBP1 as a new mediator of the crosstalk between EGF/EGFR signaling and RAC1 and a new mechanism mediating the progression of bladder cancer. The SHCBP1/RACGAP1/RAC1 interaction could therefore serve as a potential candidate for designing novel targeted therapeutic strategies for bladder cancer in clinical management.

## Materials and methods

### Cell culture and cell lines

Human bladder cancer cells (5637, T24, UMUC-3), human immortal ureteral epithelium immortalized cells (SV-HUC-1), and HEK-293T cells were purchased from the American Type Culture Collection (ATCC). 5637 and T24 cells were cultured in RPMI-1640 medium (Corning) with 10% fetal bovine serum (FBS, Gbico, USA), UMUC-3 cells were cultured in MEM medium (ScienCell, Shanghai, China) with 10% FBS, SV-HUC-1 cells were cultured in F12K medium (ScienCell, Shanghai, China) with 10% FBS, and HEK-293T cells were cultured in DMEM medium (Corning) with 10% FBS. All cell cultures were incubated at 37 °C in a humidified atmosphere with 5% CO_2_. STR certificates for cell lines were listed in Supplementary file 1–4.

### Patients and clinical samples

Twenty cases of bladder cancer samples and paired adjacent non-cancerous tissues were obtained from patients diagnosed with primary bladder cancer in the Department of Urology of the First Affiliated Hospital of Chongqing Medical University. Ethics approval required was approved from the local hospital ethic committees and a written informed consent was obtained from each patient before the study (Supplementary file 5). The specimens were frozen and stored at −80 °C until used for RNA isolation or protein extraction. The detailed information of samples was listed in Supplementary Table 1.

### Immunohistochemistry

Immunohistochemical staining of formalin-fixed, paraffin-embedded was performed as previously described [6]. All sections were assessed and scored according to the staining intensity and extent by two experienced pathologists in a blind manner, the score criterion was described specifically in the previous study [6]. Immunohistochemistry (IHC) score of >3 was defined as high expression, and ≤3 was as low expression.

### Quantitative real-time polymerase chain reaction

Total RNA extraction, reverse transcription, and real-time PCR were performed as described previously [30]. Primers were purchased from Invitrogen (Thermo Fisher Scientific, Inc.) and the sequences are exhibited in Supplementary Table 2. The cDNA was acquired by using the PrimeScript RT reagent kit (Takara, Osaka, Japan). Quantitative real-time polymerase chain reaction (RT-qPCR) was performed using SYBR Green assay (Takara) and executed by ABI 7500 Real-Time PCR System (Applied Biosystems, CA, USA). The level of ACTB was used as an internal control. Results were analyzed by the 2^−△△CT^ formula to qualify the relative mRNA expression.

### Immunoblotting

Immunoblotting analysis was performed as described before [31]. The sources of the primary antibodies were shown in supplementary methods and materials.

### Vector construction and lentivirus infection

The lentiviral vector containing SHCBP1 shRNA sequence (sh-1: CCAATTACAGTGAGTCTGATT; sh-2: GCTTGAGTGAAAGGTAGATTT), and non-effective scrambled shRNA (LV-Control, shNC) were constructed by GenePharma (Shanghai, China). Stable cells were established by transfection with these lentivirus vectors according to the manufacturer’s instructions. Antibiotic-resistant transfected cells were selected for 10 days with 1 μg/ml puromycin (Sigma, USA). Small interfering siRNA specific for RACGAP1 (siRNA-1: CTAGGACGACAAGGCAACTTT, siRNA-2: CAGGTGGATGTAGAGATCAAA) and negative control siRNA were purchased from GenePharma (Shanghai, China).

The expression vectors pCMV-Flag-RACGAP1, pCDNA3.1-HA-SHCBP1 (full length), pCDNA3.1-HA-SHCBP1 (1-428), pCDNA3.1-HA-SHCBP1 (1-548), pCDNA3.1-HA-SHCBP1 (428-672) were produced by TsingKe (Beijing, China). The mutant construct of SHCBP1 (S273A) was generated by site-directed mutagenesis and cloned into pCMV vector. Plasmids and siRNA were transfected with lipofectamine 2000 (Invitrogen, Carlsbad, CA, USA) according to the manufacturer’s protocol.

### Migration and invasion assays

Migration ability of cells was assessed using a wound-healing assay as previously described [32]. Cells were photographed at 10x magnification on a phase-contrast microscope at 0 and 24 h, and the mean % wound closure was calculated from three independent experiments.

Cell migration and invasiveness were calculated by using 24-well Transwell chambers (Corning, NY, USA) with a pore size of 8 um. Transwell upper chambers were pre-coated with (for invasion) or without (for migration) solubilized Matrigel (BD Biosciences, San Jose, CA, USA). Bladder cancer cells were pre-starved in a serum-free medium for 24 h, and then cells were seeded in the upper well of Transwell chambers and incubated for another 24 h at 37 °C in 5% CO_2_. The migrated or invaded cells in the lower surface of the filter were fixed with 3.7% formaldehyde, stained with 0.5% crystal violet. Those non-invaded cells were removed with a cotton swab. Subsequently, cells from five random fields were counted under a microscope (x100 magnification, Leica Microsystems GmbH) for each chamber. Each experiment was carried out in triplicate.

### Detection of active RAC1 and RhoA

Endogenous active guanosine triphosphate (GTP)-bound RAC1 and RhoA were pulled down using Active Rac1 Pull-Down and Detection Kit (16118, Thermo Scientific™, Pierce, USA) and Active Rho Pull-Down and Detection Kit (16116, Thermo Scientific™, Pierce, USA) according to the manufacturer’s protocol [33].

### Animal model

Four to six-week-old Balb/c nude mice were obtained from the Experimental Animal Center of Chongqing Medical University, China. For establishment of subcutaneous invasiveness model, 5637 cells (5 × 10^6^) were subcutaneously implanted into the left- or right-side dorsal flank of nude mice (*n* = 6 per group), and the mice were sacrificed for tumor excision after 4 weeks. Both the xenografted tumors and deep muscle tissues connected together were excised. Sections of samples were stained with H&E to observe the tumor boundary and structure.

For tumor metastasis assay, UMCU-3 cells (2 × 10^6^) were injected intravenously through a tail vein for each mouse. Mice were sacrificed and used for examination 6 weeks after injection. All of the procedures were approved by the animal ethics committee of our institute.

### Statistical analysis

Statistical analyses were performed in SPSS version 22.0 software (SPSS, Chicago, IL) and GraphPad 5.0 (GraphPad Software, San Diego, CA). Survival curves were plotted using Kaplan–Meier analysis and the log-rank test. Comparisons between groups were performed using the two-tailed Student’s *t*-test. One-way analysis of variance (ANOVA) was used to analyze the significance among multiple groups. A *p-*value of < 0.05 was considered statistically significant.

## Supplementary information

Supplementary Figure 1

Supplementary Figure 2

Supplementary Table 1

Supplementary Table 2

Supplementary figure legends

Supplementary methods and materials

Author contribution

Reproducibility checklist

Supplementary file 1

Supplementary file 2

Supplementary file 3

Supplementary file 4

Supplementary file 5

Supplementary file 6

## Data Availability

The datasets generated and/or analyzed during the current study are available from the corresponding author on reasonable request.
