# Supplementary figures and images for: EGF-induced nuclear translocation of SHCBP1 promotes bladder cancer progression through inhibiting RACGAP1-mediated RAC1 inactivation
Source: Cell Death Dis. 2022 Jan 10;13(1):39. doi: 10.1038/s41419-021-04479-w (PMC8748695; doi:10.1038/s41419-021-04479-w)

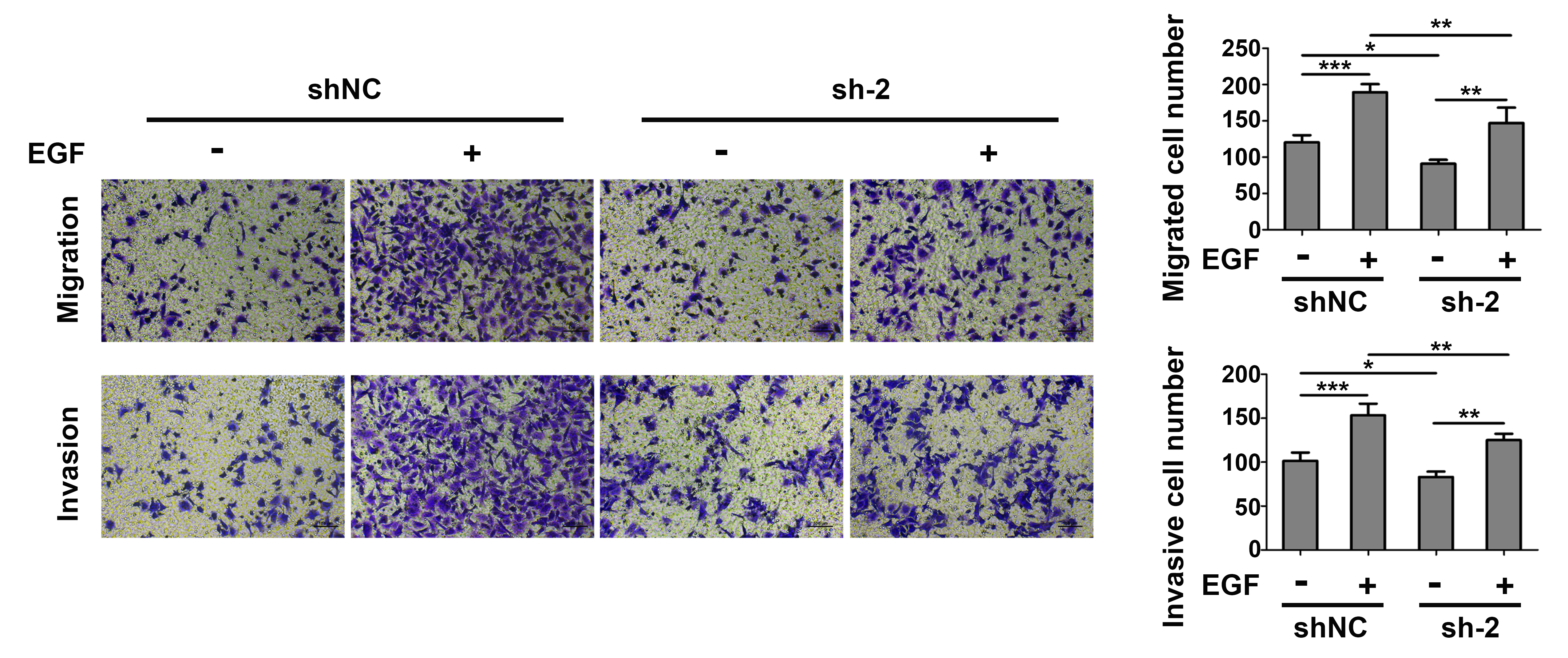

Supplement: Supplementary file 1 — Supplementary Figure 1 [file 41419_2021_4479_MOESM1_ESM.jpg]

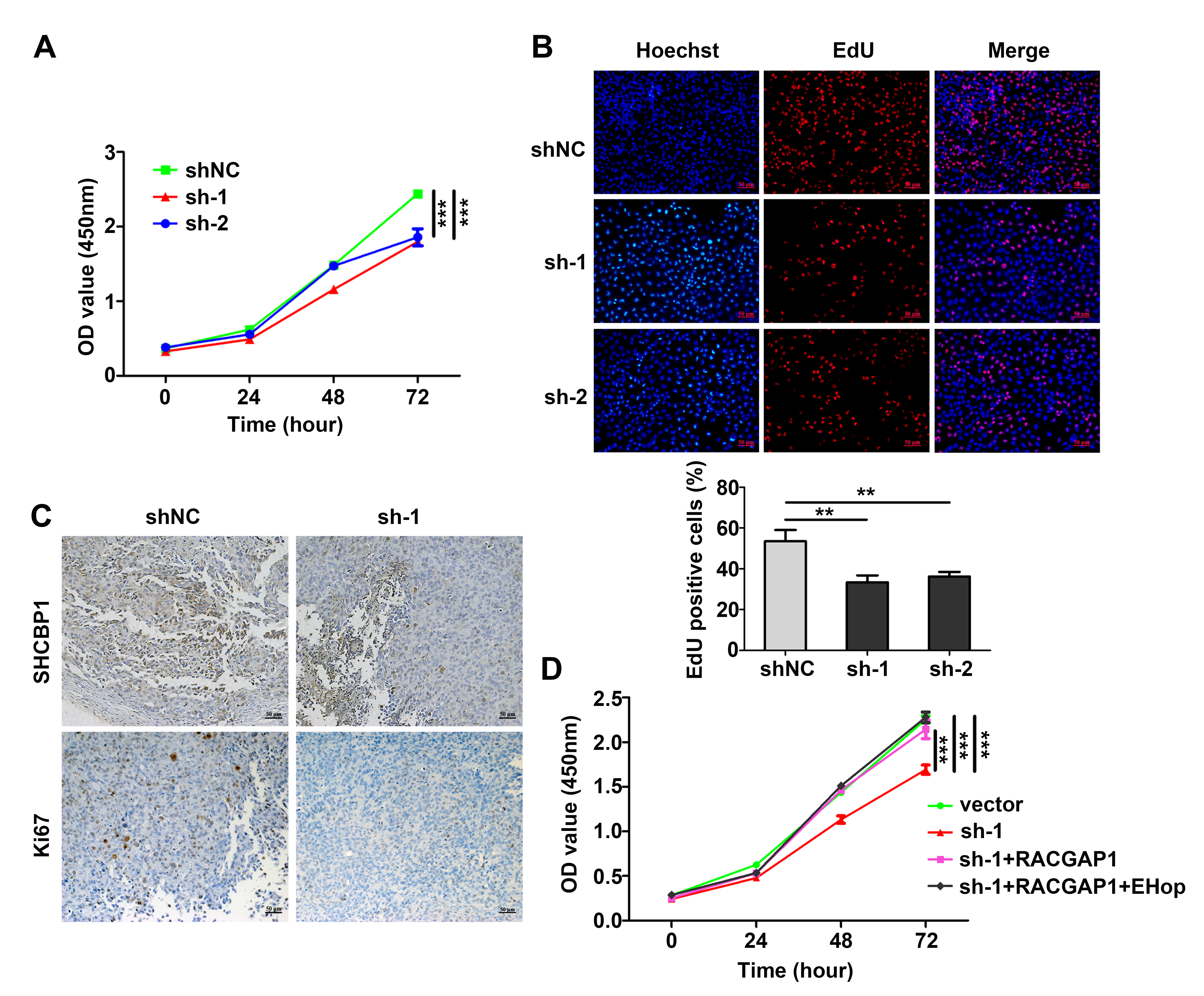

Supplement: Supplementary file 2 — Supplementary Figure 2 [file 41419_2021_4479_MOESM2_ESM.png]

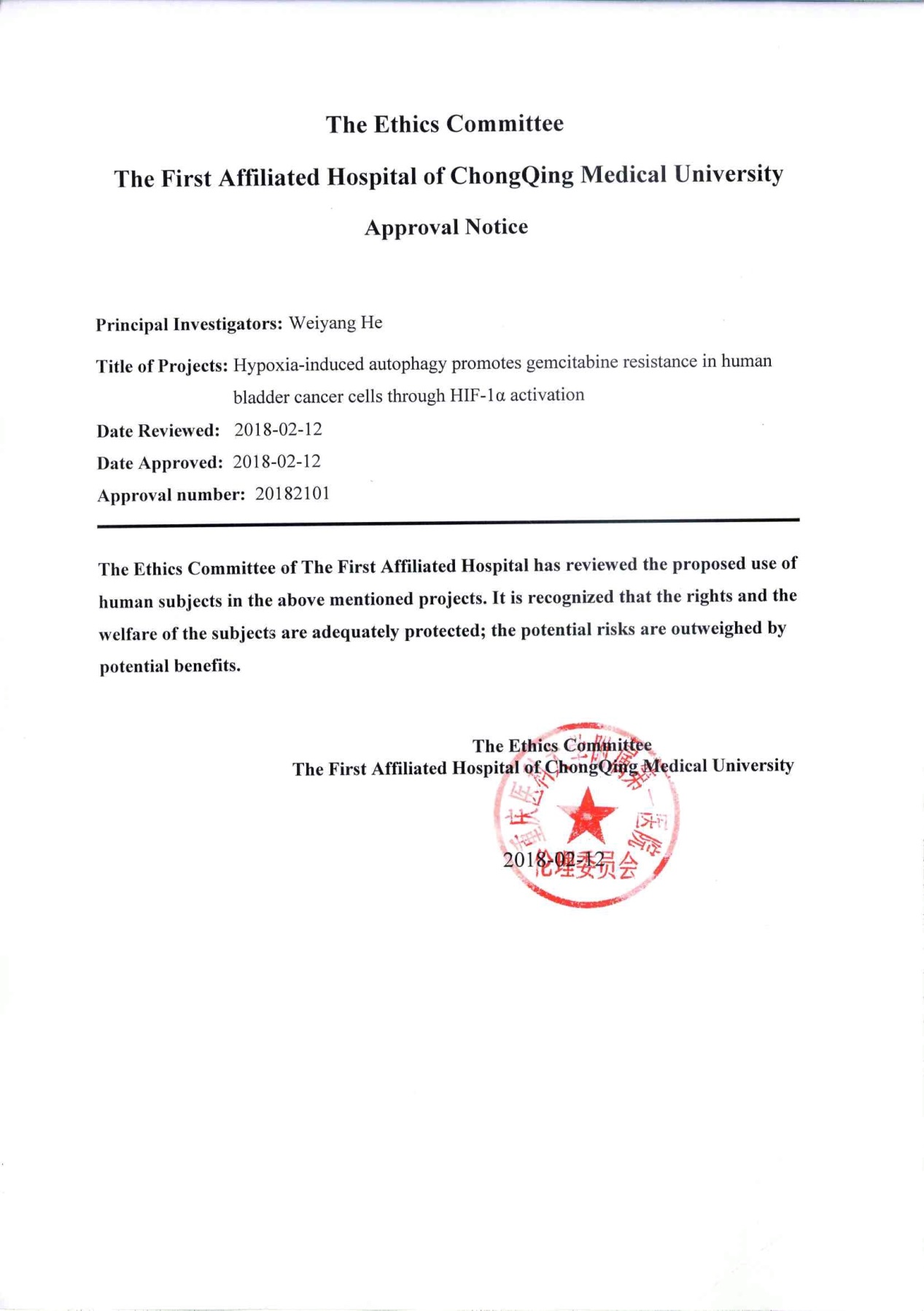

Supplement: Supplementary file 13 — Supplementary file 5 [file 41419_2021_4479_MOESM13_ESM.docx]
