# Supplementary material for: EGF-induced nuclear translocation of SHCBP1 promotes bladder cancer progression through inhibiting RACGAP1-mediated RAC1 inactivation
Source: Cell Death Dis. 2022 Jan 10;13(1):39. doi: 10.1038/s41419-021-04479-w (PMC8748695; doi:10.1038/s41419-021-04479-w)
Supplement: Supplementary file 3 — Supplementary Table 1 [file 41419_2021_4479_MOESM3_ESM.docx]

Supplementary Table 1. Clinicopathological parameters of patients.

| Characteristics | Value |
| --- | --- |
| Number of patients | 20 |
| Age (mean ± SD) | 66.31 ± 8.16 |
| Gender (Male/Female) | 12/8 |
| Pathology grade (Low/High) | 4/16 |
| Tumor size (≤ 3cm/> 3cm) | 8/12 |
| Subtype (Papillary/Non-papillary) | 11/9 |
| T stage (Ta-T_1_/T_2_-T_4_) | 5/15 |
| Lymph node metastasis (No/Yes) | 20/0 |
| TNM stage (I/II/III/IV) | 5/10/4/1 |

SD, standard deviation; TNM, tumor-node-metastasis
