# Supplementary material for: EGF-induced nuclear translocation of SHCBP1 promotes bladder cancer progression through inhibiting RACGAP1-mediated RAC1 inactivation
Source: Cell Death Dis. 2022 Jan 10;13(1):39. doi: 10.1038/s41419-021-04479-w (PMC8748695; doi:10.1038/s41419-021-04479-w)
Supplement: Supplementary file 4 — Supplementary Table 2 [file 41419_2021_4479_MOESM4_ESM.docx]

Supplementary Table 2. Primer sequence.

| Gene | Forward (5′-3′) | Reverse (5′-3′) |
| --- | --- | --- |
| SHCBP1 | GCTACCGTGATAAACCAGGTTC | AGGCTCTGAATCGCTCATAGA |
| RACGAP1 | GAAAGCAGAGACTGAGCGAAG | GTTGAATGCTGCCAGATGTGT |
| β-actin | CATGTACGTTGCTATCCAGGC | CTCCTTAATGTCACGCACGAT |
