# Supplementary material for: EGF-induced nuclear translocation of SHCBP1 promotes bladder cancer progression through inhibiting RACGAP1-mediated RAC1 inactivation
Source: Cell Death Dis. 2022 Jan 10;13(1):39. doi: 10.1038/s41419-021-04479-w (PMC8748695; doi:10.1038/s41419-021-04479-w)
Supplement: Supplementary file 5 — Supplementary figure legends [file 41419_2021_4479_MOESM5_ESM.docx]

**Supplementary Fig. 1**. Knockdown of SHCBP1 suppresses EGF-induced cell migration and invasiveness in T24 cells. **p*<0.05, **p<0.01 and ****p*<0.001.

**Supplementary Fig. 2**. SHCBP1 mediates the cell proliferation of bladder cancer cells. **A**. CCK8 assay indicating the effect of depletion of SHCBP1 using specific shRNA on cell growth in T24 cells. Data are presented as the mean ± SD, n=4. ****p*<0.001. **B**. Cell proliferation was detected using the EdU assay in T24 cells transfected with shNC, sh-1 and sh-2 (200× magnification). **C**. IHC staining of SHCBP1 and Ki67 in shNC and sh-1 group (200× magnification). **D**. CCK8 assay showing the effect of RACGAP1 overexpression and RACGAP1 overexpression combined with EHop (10 μM) on SHCBP1 loss mediated impaired cell growth in T24 cells. Data are presented as the mean ± SD, n=4. *** *p*<0.001. EHop, EHop-016.
