# Supplementary material for: EGF-induced nuclear translocation of SHCBP1 promotes bladder cancer progression through inhibiting RACGAP1-mediated RAC1 inactivation
Source: Cell Death Dis. 2022 Jan 10;13(1):39. doi: 10.1038/s41419-021-04479-w (PMC8748695; doi:10.1038/s41419-021-04479-w)
Supplement: Supplementary file 6 — Supplementary methods and materials [file 41419_2021_4479_MOESM6_ESM.docx]

**Antibodies**

anti-SHCBP1 (Proteintech, 12672-1-AP), anti-RACGAP1 (Proteintech, 13739-1-AP for immunoblotting and 66056-1-Ig for IF), anti-DYKDDDDK (Sigma, F1804), anti-p-EGFR (CST, #3777), anti-EGFR (CST, #4267), anti-p-AKT (CST, #4060), anti-AKT (CST, #4691), anti-p-ERK1/2 (CST, #4370), anti-ERK1/2 (CST, #4695), anti-Ki-67 (CST, #9449), anti-HA tag (CST, #3724), anti-β-actin (Proteintech, 66009-1-Ig), anti-phosphoserine (Abcam, ab9332), anti-GAPDH (Proteintech, 66004-1-Ig), and anti-PCNA (Santa cruz, sc-56). Anti-mouse (#7076) and anti-rabbit (#7074) peroxidase conjugated secondary antibodies were purchased from CST.

**Co-immunoprecipitation, silver staining and** **mass spectrometry**

Cells for immunoprecipitation assays were washed twice with cold PBS and lysed using lysis buffer (Beyotime, P0013) for 30 minutes. The total protein obtained by centrifugation (13,000×g, 15 min, 4°C) was incubated with primary antibodies overnight at 4 °C on a rocking platform. Appropriate amounts of Protein A/G immunoprecipitated magnetic beads (B23202, Bimake) were added to the samples followed by incubation for 6 hours at 4°C on a rocking platform. Immunocomplexes were collected by centrifugation and washed three times with lysis buffer. Finally, immunocomplexes were boiled with loading buffer and subjected to immunoblotting, silver staining, and mass spectrometry. Silver staining was performed using the Fast Silver Stain Kit (Beyotime, China) following the protocol indicated, while mass spectrometry was analyzed by Genecreate (Wuhan, China).

**Nuclear and cytoplasmic extraction**

NE-PER Nuclear and Cytoplasmic Extraction Reagents Kit (Thermo Scientific™, Pierce, USA) was used and the assays were performed according to the manufacturer’s instructions.

**Immunofluorescence (IF)**

Cells were fixed with 4% formaldehyde for 15 minutes and permeabilized with 0.5% TritionX-100 (Sigma, St Louis, MO) for 5 minutes at room temperature, blocked with 1% BSA for one hour, and then incubated with specific antibodies against SHCBP1, RACGAP1 or HA for 8 hours at 4°C, respectively. After washing with PBS, samples were labeled with goat anti-mouse IgG H&L (Alexa Fluor 488, Abcam, ab150113) and goat anti-rabbit IgG H&L (Alexa Fluor 594, Abcam, ab150080) for 1 hour in a dark place, respectively, washed with PBS, and stained with DAPI (Beyotime, C1005) for 10 minutes. Later, samples were visualized under a confocal laser scanning microscope (Olympus Fluoview 2000, Andor Belfast UK).

***In vitro* RhoGAP assay**

An in vitro GAP assay was performed using the RhoGAP ASSAY Biochem Kit (#BK105, Cytoskeleton, Denver, CO) according to the manufacturer’s protocol.

**Gene set enrichment analysis (GSEA)**

GSEA was performed using TCGA dataset including 414 patients with bladder cancer divided into high‑ and low‑SHCBP1 groups according to the median value, and executed using GSEA software 3.0 from the Broad Institute. The Hallmark gene sets (h.all.v6.1.symbols.gmt) representing specific well defined biological processes and the gene sets of canonical pathways (c2.cp.v7.2.symbols) were obtained from the Molecular Signatures Database (http://software.broadinstitute.org/gsea/msigdb/index.jsp). Gene set permutations were performed 1,000 times for each analysis to obtain normalized enrichment score (NES) used for sorting pathways enriched in each phenotype. A result was regarded as significant when nominal *p*<0.05 and false discovery rate (FDR)<0.1.

**Cell proliferation assays**

T24 cells suspensions were seeded at a density of 5×10^3^ cells per well in 96-well plates. Cell growth was detected every 24 h for 3 days using the Cell Counting Kit-8 (CCK8) assay according to the manufacturer’s instructions. After incubation at 37℃ for 1 h, absorbance of each sample at 450 nm was measured using a microplate reader (Infinite 200 PRO, TECAN, Männedorf, Switzerland).

In addition, DNA synthesis of T24 cells was also analyzed using a Cell-Light EdU Apollo 567 in vitro kit (C10310-1; Guangzhou RiboBio Co., Ltd.) according to the manufacturer’s instructions. Images were visualized and captured under a fluorescence microscope (Olympus Corporation).
