# Supplementary material for: EGF-induced nuclear translocation of SHCBP1 promotes bladder cancer progression through inhibiting RACGAP1-mediated RAC1 inactivation
Source: Cell Death Dis. 2022 Jan 10;13(1):39. doi: 10.1038/s41419-021-04479-w (PMC8748695; doi:10.1038/s41419-021-04479-w)
Supplement: Supplementary file 7 — Author contribution [file 41419_2021_4479_MOESM7_ESM.pdf]

DECLARATION OF CONTRIBUTIONS TO ARTICLE

ADMC

Manuscript Number: CDDIS-21-2977

Journal Name: *Cell Death & Disease* (the 'Journal')

Proposed Title of the Contribution:

EGF-induced nuclear translocation of SHCBP1 promotes bladder cancer progression through inhibiting RACGAP1-mediated RAC1 inactivation (the 'Contribution)

Author(s):

Hubin Yin, Chen Zhang, Zongjie Wei, Weiyang He, Ning Xu, Yingjie Xu, Tinghao Li, Ke Ren, Youlin Kuang, Xin Zhu, Fangchao Yuan, Haitao Yu, Xin Gou (the 'Authors')

For all *CDDis* articles, each person named as an author in the published version must be able to show he or she has contributed substantially to the article.

Authorship credit should be based on 1) substantial contributions to conception and design, acquisition of data, or analysis and interpretation of data; 2) drafting the article or revising it critically for important intellectual content; and 3) final approval of the version to be published. Authors should meet conditions 1, 2 and 3.

Any person who cannot be shown to have made a substantial contribution to the article cannot be listed as an author in the final version. The name of any person who is deemed to have made a minor contribution can, however, appear in the Acknowledgments section of the article.

Please complete the table below to indicate the contributions of all named authors to the manuscript.

Author Full Name:	Specification of Contribution to the Manuscript:
Hubin Yin	Design the study, perform the experiments, and manuscript preparation
Chen Zhang	Perform bioinformatics analyses, and financial support, and graph visualization
Zongjie Wei	Provide technical assistance for the main experiments
Weiyang He	Financial support, revise the original manuscript
Ning Xu	Design the study, statistical analysis
Yingjie Xu	Collecte the clinical samples and information
Tinghao Li	Performe H&E staining and analyzing histological data
Ke Ren	Provide technical assistance for the main experiments
Youlin Kuang	Perform the animal assays in vivo
FangchaoYuan	Integrate figures and graphs, and cell culture
HaitaoYu	Edit and review the manuscript, language polish
Xin Gou	Study design, main financial support, edit and review the manuscript

Please complete the table below to indicate the contributions of all named authors to the figures.

Figure 1:

Hubin Yin, Chen Zhang, Yingjie Xu, Tinghao Li

Figure 2:

Hubin Yin, Chen Zhang

Figure 3:

Hubin Yin, Youlin Kuang, Zongjie Wei, Tinghao Li

Figure 4:

Hubin Yin, Fangchao Yuan

Figure 5:

Hubin Yin

Figure 6:

Hubin Yin, Chen Zhang, Yingjie Xu

Signed for and on behalf of the Author(s):	Print Name:	Date:
Hubin Yin	Hubin Yin	10/15/2021
