# Supplementary material for: EGF-induced nuclear translocation of SHCBP1 promotes bladder cancer progression through inhibiting RACGAP1-mediated RAC1 inactivation
Source: Cell Death Dis. 2022 Jan 10;13(1):39. doi: 10.1038/s41419-021-04479-w (PMC8748695; doi:10.1038/s41419-021-04479-w)
Supplement: Supplementary file 9 — Supplementary file 1 [file 41419_2021_4479_MOESM9_ESM.pdf]

细胞遗传质量鉴定检验报告

检品名称：细胞系

检验类型：STR 基因型检验

样品编号：

表 1 样本编号

客户样本编号	公司编号
061	20161021-08

样品数量： 1

样品性状： 细胞系

检测项目： STR

送检单位： **上海中乔新舟生物技术有限公司**

检测方法： 用 Axygen 的基因组抽提试剂盒提取 DNA，采用 20- STR 扩增方案扩增，在 ABI 3730XL 型遗传分析仪上对 STR 位点和性别基因 Amelogenin 进行检测。

检验结果：

(一) 检验基本情况

表 2：样本基因型检验结果

样本编号	多等位基因	匹配细胞系	细胞库	EV 值	匹配说明
20161021-08	无	5637	DSMZ	0. 889	基本匹配

- 多等位基因指三等位及以上基因现象。
- 本次检测各细胞分型结果良好。

(二)各样本描述

- 20161021-08：该株细胞 DNA 分型在细胞系检索中找到**基本匹配**的细胞系，DSMZ 数据库显示细胞名分别为 **5637 和 5637**，细胞号分别对应 **35 和 RCB1191**。本次检测在该细胞系中**没有多等位基因**。

(三)样本分型结果

表 3：细胞 20161021-08 的 STR 位点和 Amelogenin 位点的基因分型结果

Marker	样本				细胞库信息		
	Allele1	Allele2	Allele3	Allele4	Allele1	Allele2	Allele3
D5S818	11	12			11	12	
D13S317	11	11			11	11	
D7S820	10	11			10	11	
D16S539	9	9			9	9	
VWA	18	18			18	18	
TH01	7	9			7	9	
AMEL	X	X			X	Y	
TPOX	8	8			8	9	
CSF1PO	11	11			11	11	
D12S391	20	20					
FGA	22	22					
D2S1338	25	25					
D21S11	36	36					
D18S51	16	18					
D8S1179	10	16					
D3S1358	15	17					
D6S1043	16	20					
PENTAE	10	12					
D19S433	13	15					
PENTAD	11	11					

其他说明:

(一) 分型方案及位点分布:

附表：实验方案及位点

	方案 1	方案 2	方案 3	方案 4	方案 5	方案 6
1	TH01	AMEL	TPOX	D3S1358	PENTAE	D19S433
2	D12S39<br>1	D5S818	VWA	D13S317		PENTAD
3	D7S820	D2S1338	D8S1179	D6S1043		
4	CSF1PO	D21S11		D16S539		
5	FGA	D18S51				

Applied Biosystems
GeneMapper 4.0

Cell Line Authenticaion-4

Sample File	Sample Name	Panel	OS	SQ
64_H06_CellLineAuthentication-1025.fsa	061	STR Profile 1-dup		

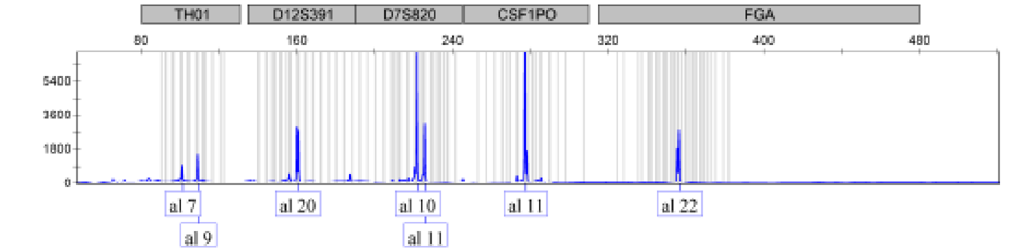

88_H11_CellLineAuthentication-1025.fsa	061	STR Profile 2-dup		

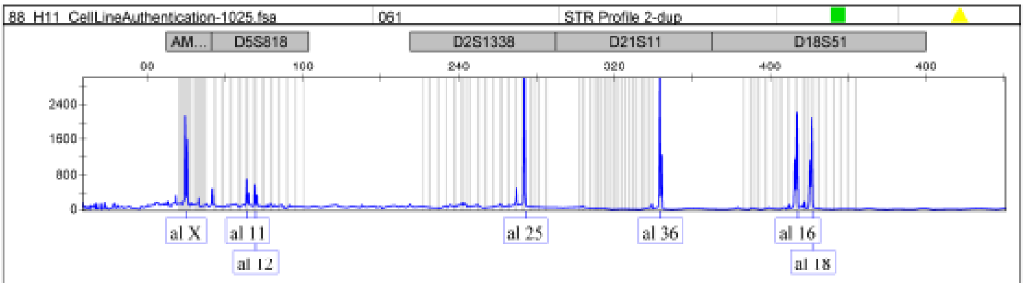

16_H02_CellLineAuthentication-1026.fsa	061	STR Profile 3-dup		

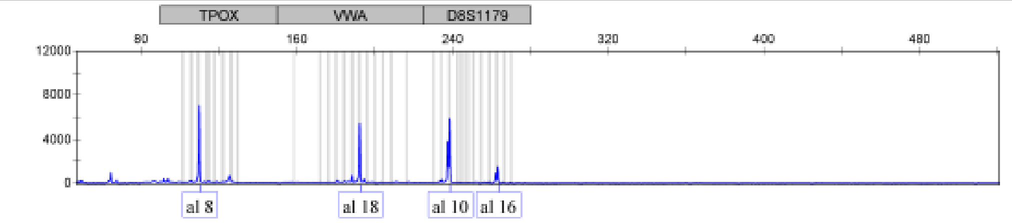

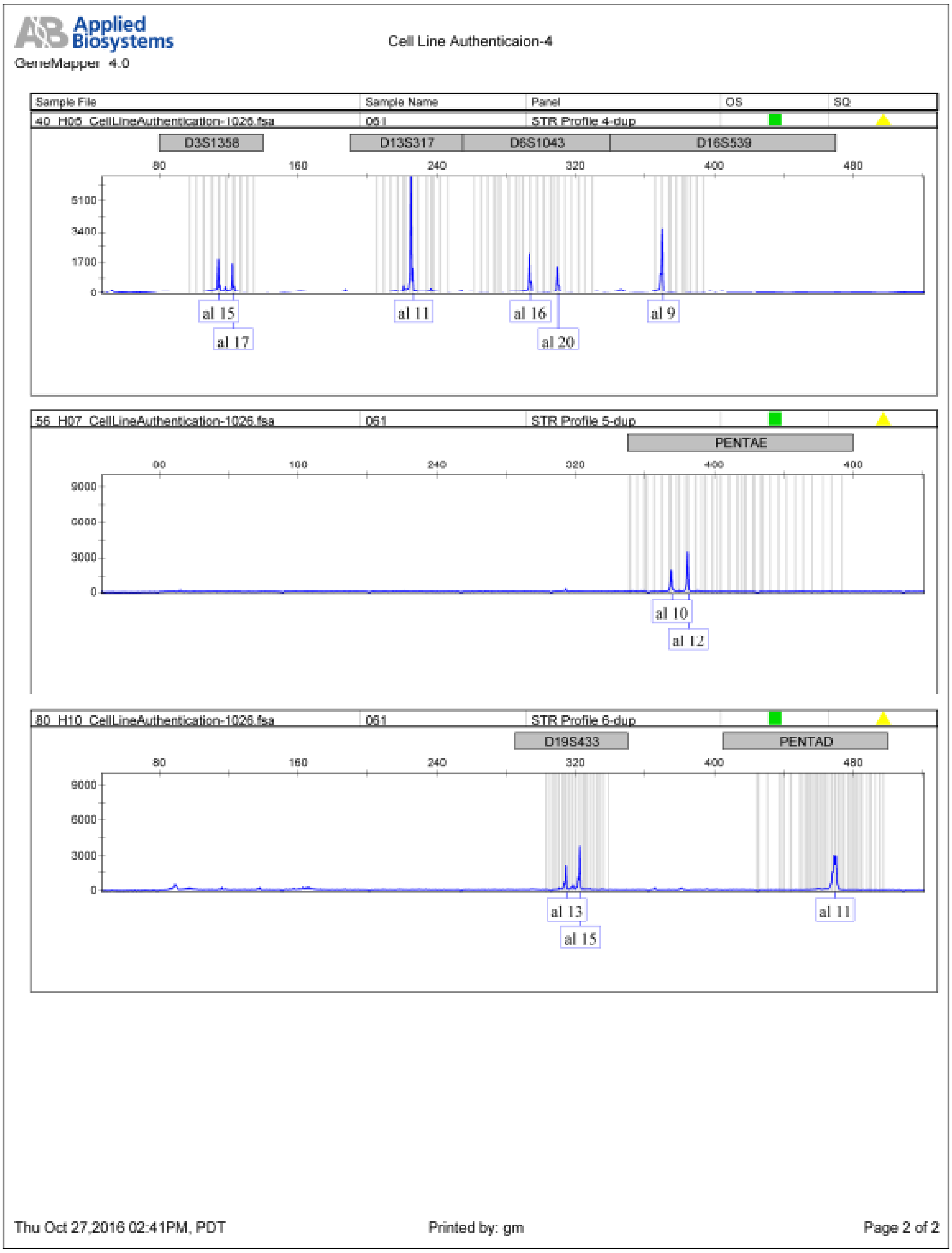

签发日期:
2016 年 10 月
