# Supplementary material for: EGF-induced nuclear translocation of SHCBP1 promotes bladder cancer progression through inhibiting RACGAP1-mediated RAC1 inactivation
Source: Cell Death Dis. 2022 Jan 10;13(1):39. doi: 10.1038/s41419-021-04479-w (PMC8748695; doi:10.1038/s41419-021-04479-w)
Supplement: Supplementary file 10 — Supplementary file 2 [file 41419_2021_4479_MOESM10_ESM.pdf]

Cell Line T24 STR Profile Report

Cell Line Designation: T24

Date Sample Received: Oct 20th, 2017

Report Date: Oct 31th, 2017

Methodology: Twenty short tandem repeat (STR) loci plus the gender determining locus,Amelogenin, were amplified using the commercially available PowerPlex® 21 System from Promega Corporation. The amplified products were processed using the Applied Biosystems 3730xl DNA Analyzer. Data were analyzed using GeneMapper 5.0 software (Applied Biosystems). Appropriate positive and negative controls were run and confirmed for each sample submitted.

Data Interpretation: Cell lines were authenticated using Short Tandem Repeat (STR) analysis as described in 2012 in ANSI Standard (ASN-0002) by the ATCC Standards Development Organization (SDO) and in Capes-Davis et al., Match criteria for human cell line authentication: Where do we draw the line? Int J Cancer.2013;132(11):2510-9.

Genechem

GENECHEM performs STR Profiling following ISO 9001:2008 and ISO/IEC 17025:2005 quality standards.

There are no warranties with respect to the services or results supplied, express or implied, including, without limitation, any implied warranty of merchantability or fitness for a particular purpose. GENECHEM is not liable for any damages or injuries resulting from receipt and/or improper, inappropriate, negligent or other wrongful use of the test results supplied, and/or from misidentification, misrepresentation, or lack of accuracy of those results. Your exclusive remedy against GENECHEM and those supplying materials used in the services for any losses or damage of any kind whatsoever, whether in contract, tort, or otherwise, shall be, at GENECHEM 's option, refund of the fee paid for such service or repeat of the service.

- Shanghai Genechem Co., Ltd.
- Add：NO.332, Aidisheng Road, Zhangjiang Hi-Tech Park, Shanghai 201203 P.R. China Tel : (86) 21 51320189 Fax : (86) 21 51370635

TEST RESULTS:

Test Results for Submitted Sample		Reference Database Profile
**Loci**	**Query Profile: T24**	**Database Profile: T24**
**Amelogenin**	X	X
**D13S317**	12	12
**D16S539**	9	9
**CSF1PO**	10 12	10 12
**TH01**	6	6
**vWA**	17	17
**D7S820**	10 11	10 11
**D5S818**	10 12	10 12
**TPOX**	8 11	8 11
**D3S1358**	16	
**D1S1656**	12 15	
**D6S1043**	11	
**Penta E**	7 10	
**D18S51**	16 18	
**D2S1338**	20 23	
**Penta D**	11 15	
**D8S1179**	14	
**D12S391**	18	
**D19S433**	14	
**FGA**	17 22	
**D21S11**	29	
Note: The top nine Loci (8 core STR loci plus Amelogenin) can be made public to verify cell identity. In order to protect the identity of the donor, ***please do not publish*** *the allele calls from all the STR loci tested.*		

Percent Match: **100%**

The submitted profile is an exact match for the following human cell line(s) in the reference database (8 core loci plus Amelogenin): **T24**

Explanation of Test Results

Cell lines with ≥80% match are considered to be related; i.e., derived from a common ancestry. Cell lines with between a 55% to 80% match require further profiling for authentication of relatedness. Reference Database Profile was obtained from ExPASy, DSMZ, ATCC or China National Infrastructure of Cell Line Resource STR database.

Genechem

Applied Biosystems
GeneMapper Software 5

0810-final

Sample File	Sample Name	Panel	OS	SQ
116_G06.fsa	G06	PowerPlex_21_POP7_Panels_v2.0		

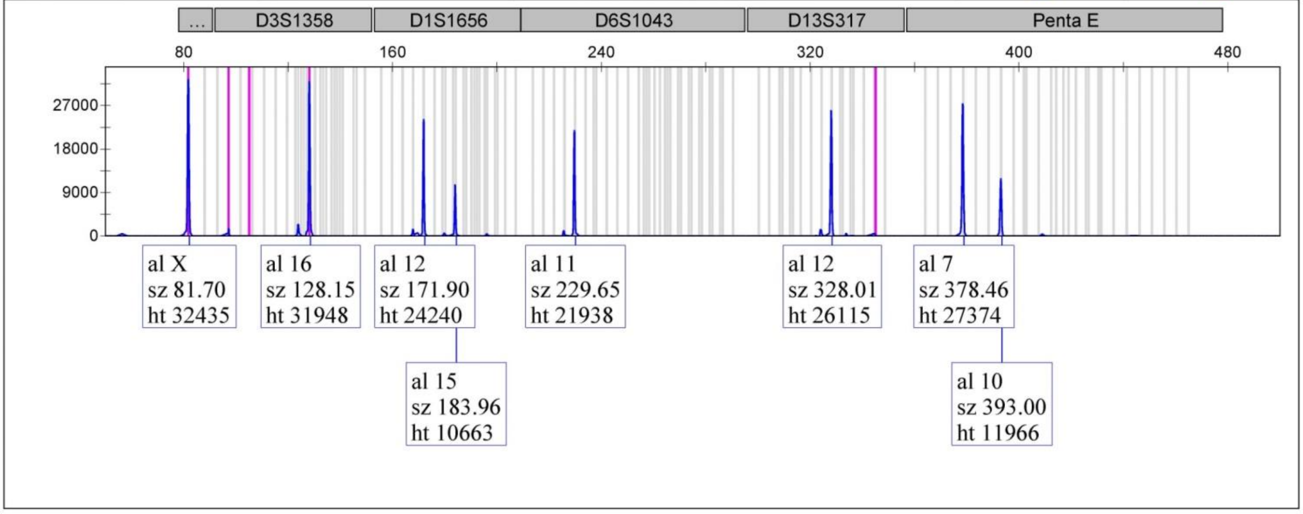

116_G06.fsa	G06	PowerPlex_21_POP7_Panels_v2.0		

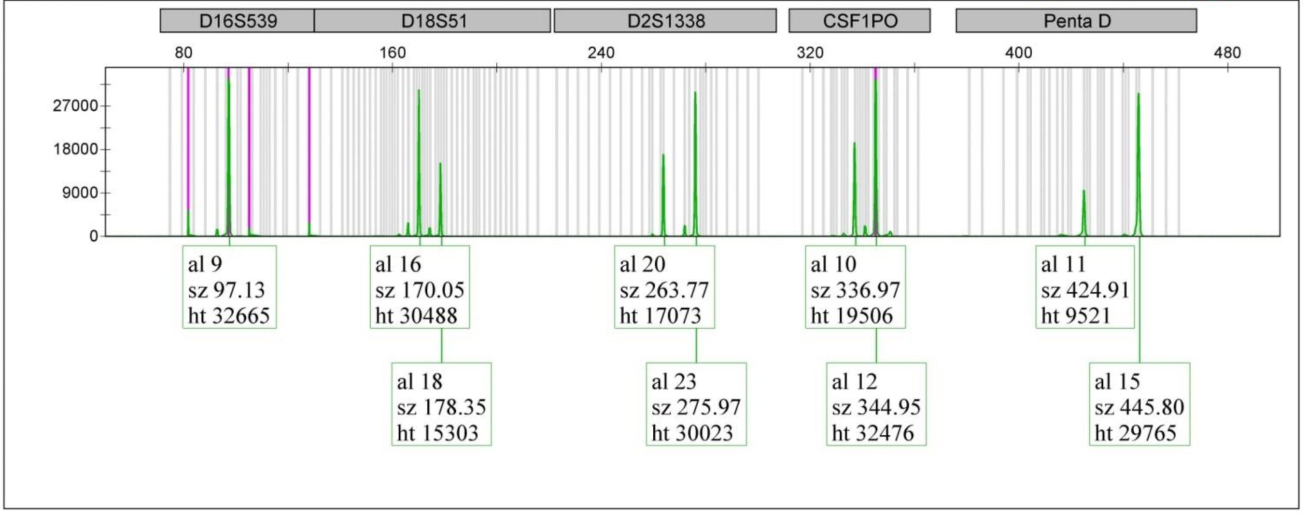

Wed Oct 25,2017 03:40PM, CST   Printed by: gm   Page 1 of 2

Applied Biosystems
GeneMapper Software 5

0810-final

Sample File	Sample Name	Panel	OS	SQ
116_G06.fsa	G06	PowerPlex_21_POP7_Panels_v2.0		

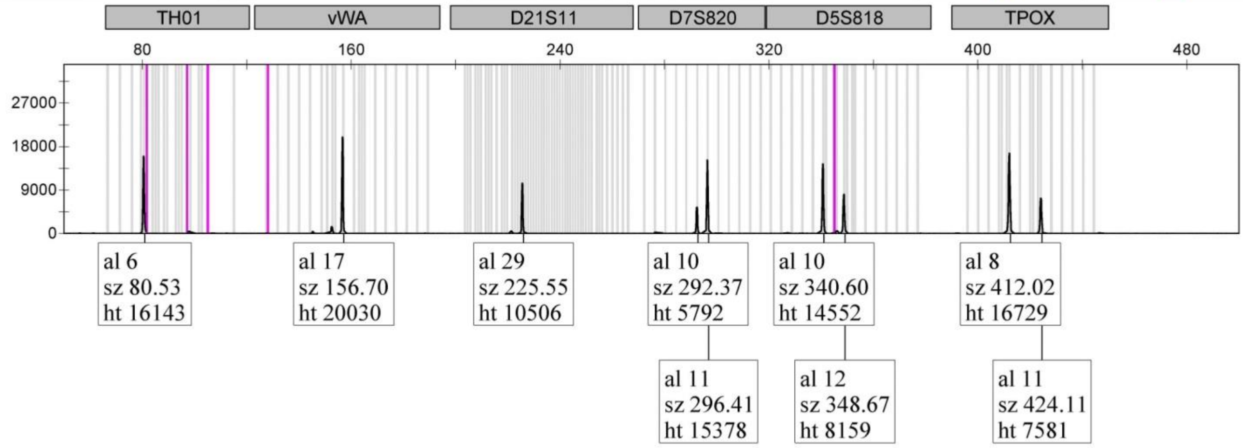

116_G06.fsa	G06	PowerPlex_21_POP7_Panels_v2.0		

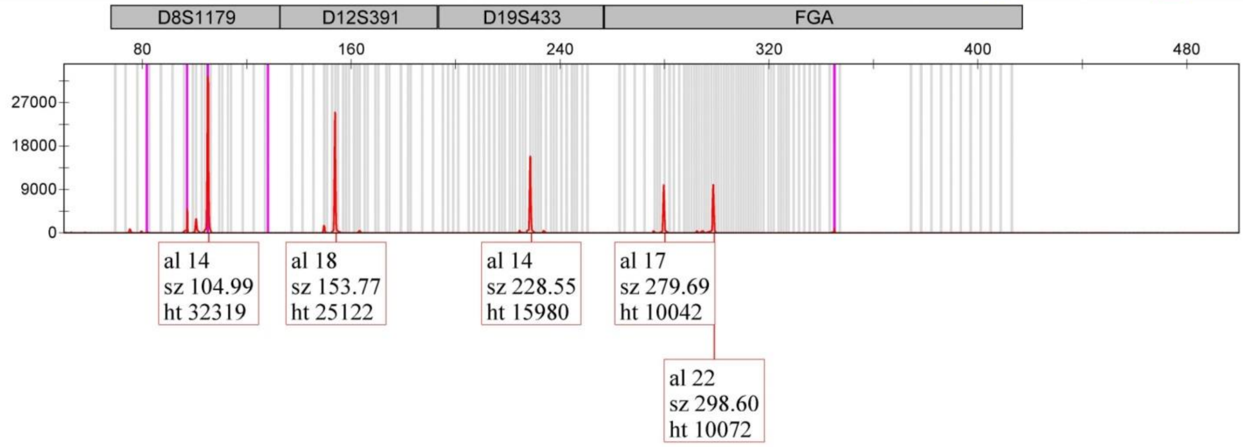

Wed Oct 25,2017 03:40PM, CST Printed by: gm Page 2 of 2
