# Supplementary material for: EGF-induced nuclear translocation of SHCBP1 promotes bladder cancer progression through inhibiting RACGAP1-mediated RAC1 inactivation
Source: Cell Death Dis. 2022 Jan 10;13(1):39. doi: 10.1038/s41419-021-04479-w (PMC8748695; doi:10.1038/s41419-021-04479-w)
Supplement: Supplementary file 11 — Supplementary file 3 [file 41419_2021_4479_MOESM11_ESM.pdf]

细胞遗传质量鉴定检验报告

检品名称：细胞系

检验类型：STR 基因型检验

样品编号：

表 1 样本编号

客户样本编号	公司编号
192	20170315-18

样品数量： 1

样品性状： 细胞系

检测项目： STR

送检单位： **上海中乔新舟生物技术有限公司**

检测方法： 用 Axygen 的基因组抽提试剂盒提取 DNA，采用 20- STR 扩增方案扩增，在 ABI 3730XL 型遗传分析仪上对 STR 位点和性别基因 Amelogenin 进行检测。

检验结果：

(一)检验基本情况

表 2：样本基因型检验结果

	**多等位基因**	**匹配细胞系**	**细胞库**	**EV 值**	**匹配说明**
20170315-18	无	UM-UC-3	DSMZ	1	完全匹配

- 多等位基因指三等位及以上基因现象。
- 本次检测各细胞分型结果良好。

(二)各样本描述

- 20170315-18：该株细胞 DNA 分型在细胞系检索中找到完全匹配的细胞系，DSMZ 数据库显示细胞名为 **UM-UC-3**，细胞号对应 **CRL-1749**，本次检测在该细胞系中**没有发现多等位基因**。

(三)样本分型结果

表 3：细胞 20170315-18 的 STR 位点和 Amelogenin 位点的基因分型结果

Marker	样本				细胞库信息		
	Allele1	Allele2	Allele3	Allele4	Allele1	Allele2	Allele3
D5S818	12	12			12	12	
D13S317	8	8			8	8	
D7S820	8	9			8	9	
D16S539	8	9			8	9	
VWA	17	17			17	17	
TH01	6	9			6	9	
AMEL	X	X			X	X	
TPOX	10	10			10	10	
CSF1PO	10	11			10	11	
D12S391	22	22					
FGA	20	21					
D2S1338	23	23					
D21S11	31	31					
D18S51	14	14					
D8S1179	13	13					
D3S1358	17	18					
D6S1043	11	13					
PENTAE	12	12					
D19S433	14	15					
PENTAD	13	13					

其他说明:

(一) 分型方案及位点分布:

附表：实验方案及位点

	方案 1	方案 2	方案 3	方案 4
1	TH01	TPOX	D3S135<br>8	AMEL
2	D12S39<br>1	VWA	D13S31<br>7	D5S818
3	D7S820	D8S1179	D6S104<br>3	D2S1338
4	CSF1PO	PENTAD	D16S53<br>9	D21S11
5	FGA		D19S43<br>3	D18S51
6	PENTAE			

中乔新舟
CELL RESEARCH

Certificate of STR Analysis

Applied Biosystems
GeneMapper 4.0

CellLineAuthentication-11

Sample File	Sample Name	Panel	OS	SQ
28_D04_CellLineAuthentication-1-0322.FSA	192	STR Profile 1-human		

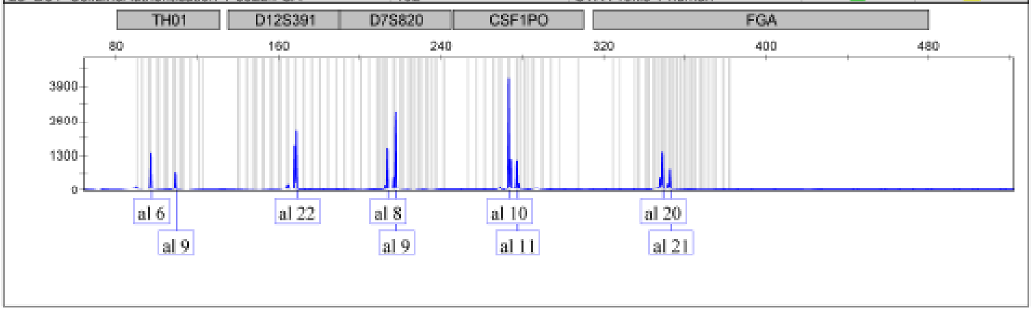

Sample File	Sample Name	Panel	OS	SQ
28_D04_CellLineAuthentication-1-0322.FSA	192	STR Profile 1-human		

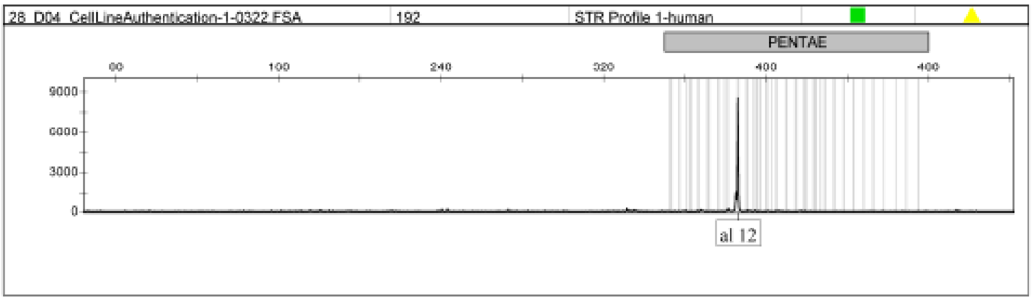

Sample File	Sample Name	Panel	OS	SQ
84_D11_CellLineAuthentication-1-0322.FSA	192	STR Profile 2-human		

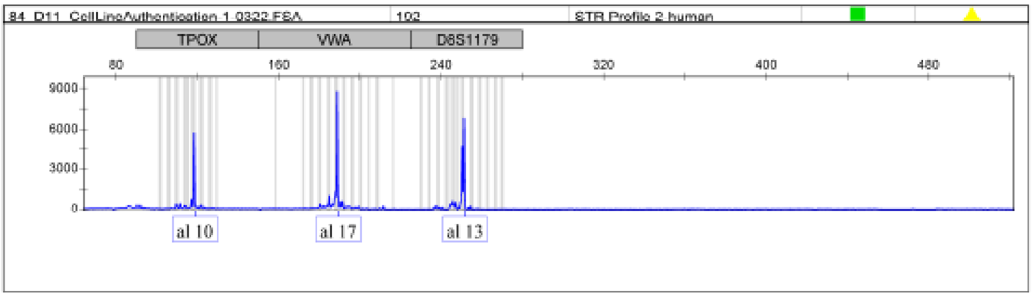

Sample File	Sample Name	Panel	OS	SQ
84_D11_CellLineAuthentication-1-0322.FSA	192	STR Profile 2-human		

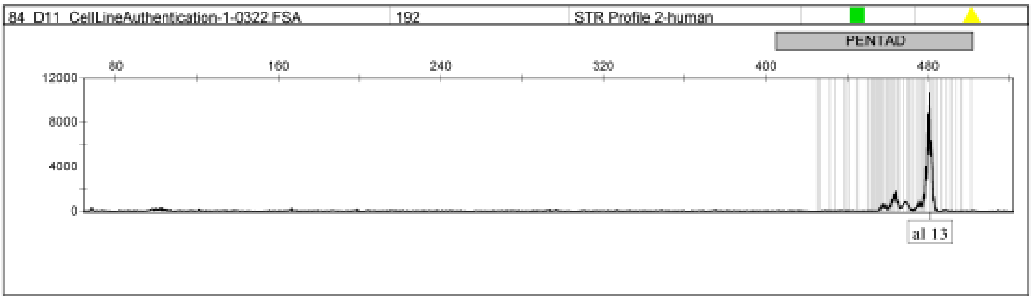

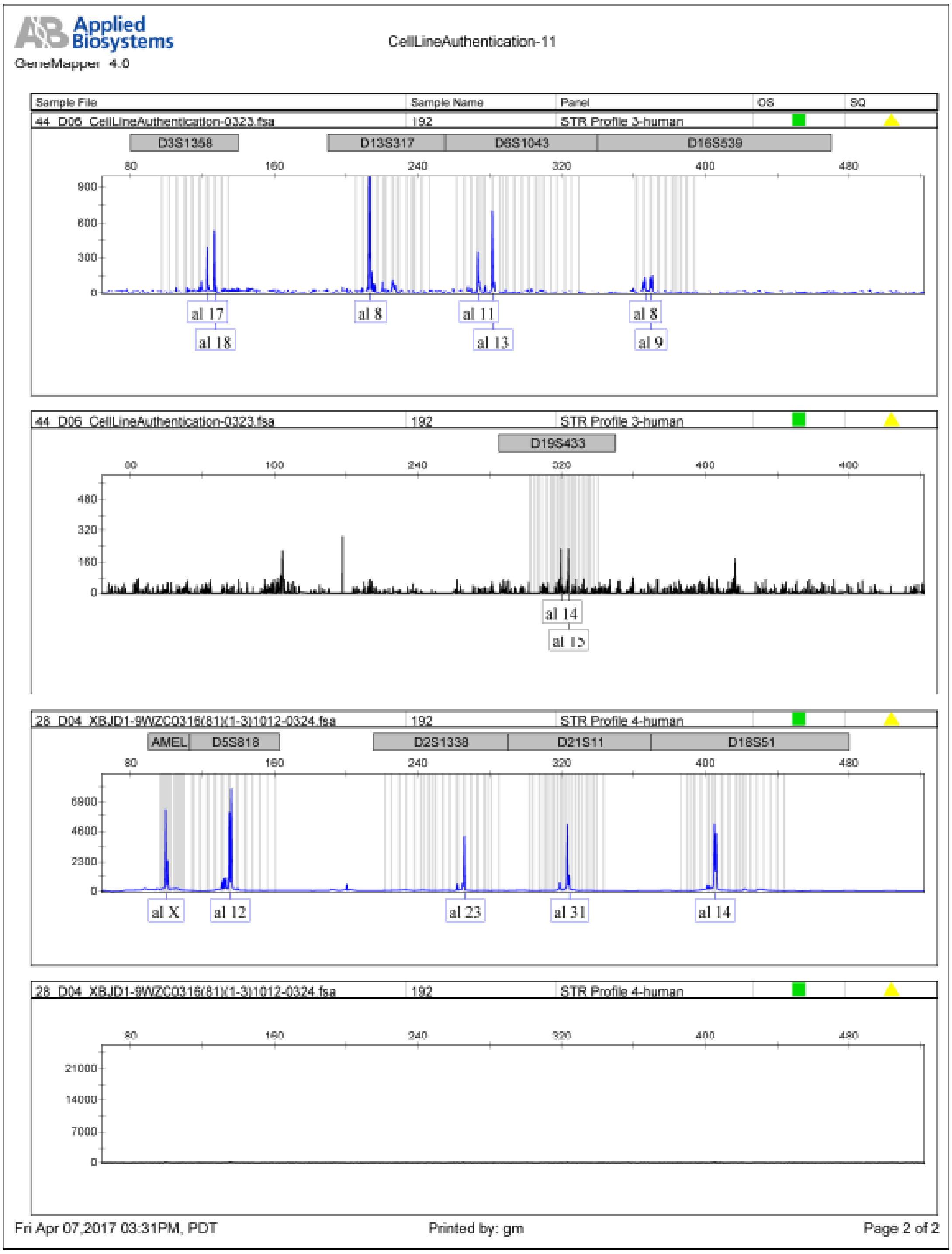

签发日期：
2017 年 05 月
