# Supplementary material for: EGF-induced nuclear translocation of SHCBP1 promotes bladder cancer progression through inhibiting RACGAP1-mediated RAC1 inactivation
Source: Cell Death Dis. 2022 Jan 10;13(1):39. doi: 10.1038/s41419-021-04479-w (PMC8748695; doi:10.1038/s41419-021-04479-w)
Supplement: Supplementary file 14 — Supplementary file 6 [file 41419_2021_4479_MOESM14_ESM.pdf]

Editing Certificate

This document certifies that the manuscript

EGF-induced nuclear translocation of SHCBP1 promotes bladder cancer progression through inhibiting RACGAP1-mediated RAC1 inactivation

prepared by the authors

Hubin Yin, Chen Zhang, Zongjie Wei, Weiyang He, Ning Xu, Yingjie Xu, Tinghao Li, Ke Ren, Youlin Kuang, Xin Zhu, Fangchao Yuan, Haitao Yu, Xin Gou

was edited for proper English language, grammar, punctuation, spelling, and overall style by one or more of the highly qualified native English speaking editors at AJE.

This certificate was issued on **July 23, 2021** and may be verified on the AJE website using the verification code **3643-4392-1BF2-F209-74CF**.

Neither the research content nor the authors' intentions were altered in any way during the editing process. Documents receiving this certification should be English-ready for publication; however, the author has the ability to accept or reject our suggestions and changes. To verify the final AJE edited version, please visit our verification page at aje.com/certificate. If you have any questions or concerns about this edited document, please contact AJE at support@aje.com.

AJE provides a range of editing, translation, and manuscript services for researchers and publishers around the world.
For more information about our company, services, and partner discounts, please visit aje.com.
